# RI Graph Sampling: A Hybrid Graph Sampling Method for Large-Scale Botnet Detection

**DOI:** 10.3390/s26196154

**Published:** 2026-09-28

**Authors:** Jia Chai, Yanping Shen, Wuxin Tian, Xiaoling Sun, Liqin Tian

**Affiliations:** School of Computer and Information Security, University of Emergency Management, Sanhe 065201, China; chaijia2002@163.com (J.C.); 15102821950@163.com (W.T.); sunxiaoling@cidp.edu.cn (X.S.); tianliqin@ncist.edu.cn (L.T.)

**Keywords:** botnet detection, feature extraction, graph convolutional network, graph sampling

## Abstract

Large-scale botnet detection using graph neural networks (GNNs) often requires subgraph sampling to reduce computational and memory costs. However, conventional sampling strategies may fail to simultaneously preserve the global topology and local community structure of the original graph, resulting in structural information loss. To address this issue, this paper proposes RI Graph Sampling (RIGS), a hybrid graph sampling method that probabilistically combines Rank Degree (RD) sampling with Improved Forest Fire Sampling based on PageRank (IFFST-PR). By exploiting the complementary structural preferences of these two strategies, RIGS preserves structurally important nodes and local community information while controlling computational overhead. RIGS is further integrated into the GraphSAINT training framework, where sampling normalization is employed to reduce the estimation bias introduced by stochastic subgraph sampling. When combined with a Graph Convolutional Network (GCN), the proposed framework alleviates the neighbor explosion problem and improves training efficiency while maintaining competitive detection performance. Experiments on the CTU-13 and NCC-2 datasets demonstrate that the proposed framework reduces training time and memory consumption while achieving competitive botnet detection performance. Structural preservation analysis further shows that RIGS provides a favorable balance between global topology and local community structure. Overall, the proposed framework achieves a favorable trade-off among detection performance, computational efficiency, and structural preservation, supporting its applicability to large-scale botnet detection.

## 1. Introduction

In recent years, botnets have become a major threat to network security because of their increasingly complex structures and expanding scale. Therefore, developing botnet detection systems with high detection accuracy and low latency is of considerable practical importance [1]. Graph neural networks (GNNs) can effectively model communication relationships among network entities and have demonstrated strong capabilities in capturing both structural correlations and behavioral characteristics in communication graphs. Consequently, they have been increasingly applied to botnet detection tasks [2]. However, when applied to large-scale communication graphs, GNNs often suffer from the “neighbor explosion” problem. As the number of GNN layers increases, the number of neighboring nodes involved in message aggregation can grow rapidly, leading to substantial computational and memory overhead and limiting training efficiency and practical deployment [3]. To alleviate this problem, GraphSAINT performs GNN training on sampled subgraphs, thereby restricting the aggregation scope and reducing computational costs.

Despite its computational efficiency, GraphSAINT primarily relies on stochastic sampling and does not explicitly account for the structural characteristics of the original graph when selecting nodes or edges. Consequently, the sampled subgraphs may fail to simultaneously preserve important global topological properties and local community structures, thereby reducing their ability to represent the structural information contained in the original graph [4]. In botnet detection, such structural information is particularly important because abnormal communication patterns and structurally important nodes may provide useful evidence for identifying botnet activity. Loss of this information during sampling may therefore degrade the subsequent detection performance.

Existing topology-aware graph sampling strategies exhibit different and potentially complementary structural preferences. Rank Degree (RD) sampling [5] prioritizes high-degree nodes and therefore tends to retain structurally important nodes and global connectivity. In contrast, Improved Forest Fire Sampling based on PageRank (IFFST-PR) [6] emphasizes community-oriented exploration and is better suited to preserving local structural information. Meanwhile, GraphSAINT employs sampling normalization to reduce estimation bias caused by unequal node inclusion probabilities. These complementary properties motivate the development of RI Graph Sampling (RIGS), which integrates RD-based and IFFST-PR-based node selection strategies within a unified probabilistic sampling process while retaining the GraphSAINT normalization mechanism during model training.

To address the limitations of conventional random subgraph sampling, RIGS constructs each sampled subgraph through an iterative hybrid sampling process. At each sampling step, either the RD-based or IFFST-PR-based node selection strategy is activated according to a predefined probability. The RD branch favors structurally important nodes based on degree information, whereas the IFFST-PR branch performs PageRank-guided local exploration to preserve community structure. The selected node and its associated edges are then incorporated into the current sampled subgraph, and this process continues until the predefined sampling budget is reached. Through this node-level hybridization, RIGS aims to balance global topological preservation with local community retention.

To mitigate the estimation bias introduced by stochastic hybrid sampling, RIGS adopts the GraphSAINT normalization mechanism during model training. Based on the proposed sampling method, we further develop a GNN-based botnet detection framework. Network traffic records are first transformed into a communication graph containing node-level traffic features and topological relationships. RIGS is then applied to generate structurally representative sampled subgraphs, which are used to train a GCN for botnet node classification.

Different from the GraphSAINT that relies on random sampling strategies, RI Graph Sampling probabilistically integrates RD and IFFST-PR. During subgraph construction, the sampling strategy is dynamically selected [7] according to a probability parameter α, where RD is selected with probability 1−α and IFFST-PR is selected with probability α. The selected strategy is then used to iteratively expand the sampled subgraph until the target sampling size is reached.

Based on RIGS, this paper further develops a graph neural network-based botnet detection framework. First, network traffic records are transformed into a communication graph that incorporates both traffic features and topological relationships. Then, RIGS is employed to generate sampled subgraphs that preserve both global topological characteristics and local community structures. Finally, a Graph Convolutional Network (GCN) is trained on the sampled subgraphs using the GraphSAINT normalization framework to accurately identify botnet nodes.

The main contributions of this paper are as follows:

1. We propose RI Graph Sampling (RIGS), a hybrid subgraph sampling method that integrates the degree-oriented structural preference of RD with the community-oriented exploration mechanism of IFFST-PR. The proposed method is designed to preserve both global topological characteristics and local community structures during subgraph sampling.
2. We develop a RIGS-based botnet detection framework for large-scale communication graphs. By restricting GNN computation to sampled subgraphs and incorporating GraphSAINT normalization, the framework reduces computational and memory overhead while maintaining competitive detection performance.
3. Extensive experiments on the CTU-13 [8] and NCC-2 [9] datasets evaluate RIGS in terms of detection performance, computational efficiency, and structural preservation. The results demonstrate a favorable trade-off among these three aspects compared with representative graph sampling methods.

## 2. Related Work

Traditional botnet detection methods primarily rely on traffic behavioral features or topological characteristics. Udiyono et al. [10], for example, proposed a botnet detection model based on the joint analysis of DNS and HTTP traffic. The method extracts multiple DNS- and HTTP-related features from network traffic and combines them with machine learning algorithms for classification. The experimental results reported an accuracy of approximately 86%, demonstrating the ability of the method to distinguish botnet traffic from benign traffic. Alharbi et al. [11] proposed a graph-based machine learning approach for botnet detection, in which network communication relationships are represented as a graph and structural features, such as node degree and centrality, are extracted for classification. Their experimental results on the CTU-13 and IoT-23 datasets reported detection accuracies of up to approximately 90%. Despite these advances, conventional machine learning approaches still have several limitations. Methods relying primarily on traffic features may become less effective when attackers deliberately disguise their traffic behavior, whereas topology-based approaches often depend on manually designed structural features, which may limit their scalability and generalization capability [12].

With the rapid development of graph neural networks (GNNs), graph-based deep learning has increasingly been applied to botnet detection. Meng et al. [12] proposed a GNN-based botnet detection model that represents large-scale network communication as a graph and jointly learns traffic and topological characteristics. The authors reported higher detection rates and lower false-positive rates than conventional methods across multiple synthetic and real botnet topologies, together with improved generalization performance. However, GNNs may suffer from the “neighbor explosion” problem when applied to large-scale graphs or deeper architectures. As the number of GNN layers increases, the number of neighboring nodes involved in message aggregation can grow rapidly, resulting in substantial computational and memory overhead and reduced training efficiency [13].

To alleviate the neighbor explosion problem, various graph sampling strategies have been developed to reduce the computational cost of GNN training. According to their sampling mechanisms, these approaches can generally be categorized into node-wise, layer-wise, and subgraph-based sampling methods [4]. Node-wise approaches, such as GraphSAGE proposed by Hamilton et al. [14], restrict the aggregation scope by sampling a fixed subset of neighboring nodes at each layer. Layer-wise approaches, such as S-GCN proposed by Chen et al. [15], reduce computational overhead by limiting the number of sampled nodes at each layer and reusing sampled neighbors across the computation graph. FastGCN [16] further reduces inter-layer dependencies by independently sampling nodes for individual GNN layers.

Subgraph-based sampling methods have also attracted considerable attention. Zeng et al. [17] proposed a subgraph-based GCN training method in which multiple subgraphs are sampled and used for mini-batch optimization. Chiang et al. [18] introduced ClusterGCN, which partitions the original graph into clusters and randomly selects one or more clusters to construct training subgraphs. Although these approaches alleviate neighbor explosion to some extent, graph partitioning or subgraph extraction may disrupt the connectivity of the original graph and introduce training bias, particularly for deep GCNs or sparse graphs [19].

GraphSAINT, proposed by Zeng et al. [3], performs GNN training directly on sampled subgraphs and employs normalization to correct sampling-induced estimation bias. It provides three representative sampling strategies: random node sampling, random edge sampling, and random walk sampling. These strategies are relatively simple to implement and provide high computational efficiency for large-scale graph learning. Shi et al. [20] further proposed Local Message Compensation (LMC), which improves gradient estimation during subgraph-based training by compensating for messages from nodes outside the sampled mini-batch during forward and backward propagation. Yin et al. [21] combined GraphSAINT with the Graph Isomorphism Network (GIN) for IoT botnet detection, using GIN to improve the representation of graph structural information.

Despite its computational advantages, GraphSAINT primarily relies on stochastic sampling and does not explicitly prioritize structural information during node or edge selection. Consequently, the resulting subgraphs may not adequately preserve both the global topology and local community structure of the original graph, potentially limiting their structural representativeness [22].

To improve the structural representativeness of sampled subgraphs, a variety of topology-aware sampling strategies have been investigated. Methods based on global structural properties, such as Degree Importance Sampling (IS) [23], Partial Induced Edge Sampling (PIES) [24], and Rank Degree (RD) sampling [5], tend to preserve structurally important nodes and global topological characteristics. However, they may provide limited preservation of local community integrity. In contrast, community-oriented methods, such as Expansion Sampling [25], Community-Driven Sampling (CBS) [26], and Improved Forest Fire Sampling based on PageRank (IFFST-PR) [6], are better suited to retaining local community structures but may provide less explicit preservation of global topology.

Xu et al. [7] proposed a hybrid sampling method that combines random walk and random jump mechanisms. In this method, the sampling process performs random walk with probability 1−α and random jump with probability α. While sufficiently sampling the local communities of nodes, the jump mechanism is used to avoid being trapped in local regions, thereby improving the overall quality of the sampled graph. Subsequent studies further improved the hybrid sampling mechanism. Qi X. [27] introduced an adaptive jump mechanism based on node degree within the hybrid sampling framework. By introducing parameter C, the jump probability is associated with node degree, assigning higher jump probabilities to low-degree nodes and higher random walk probabilities to high-degree nodes. However, this method still has certain limitations. Such methods mainly rely on node degree information to design the sampling strategy and lack the utilization of higher-order structural information in graphs, such as community structures. Moreover, sampling strategies based on random walk and random jump make it difficult to preserve the structural integrity of the sampled subgraphs.

In summary, existing graph sampling methods exhibit different structural preferences, and preserving global topology and local community structure simultaneously remains challenging. Developing a sampling strategy that effectively balances these two types of structural information while maintaining computational efficiency is therefore an important problem for large-scale botnet detection. This challenge motivates the hybrid sampling framework proposed in this study.

## 3. Background

This section introduces three techniques underlying the proposed RIGS framework: Rank Degree (RD) sampling, Improved Forest Fire Sampling based on PageRank (IFFST-PR), and GraphSAINT sampling normalization. RD and IFFST-PR are existing graph sampling methods with different structural preferences, whereas GraphSAINT normalization is used to correct the estimation bias introduced by stochastic subgraph sampling. These techniques provide the methodological background for the proposed hybrid sampling framework presented in Section 4.

### 3.1. Rank Degree (RD)

The basic principle of Rank Degree (RD) sampling [5] is to prioritize nodes with high degree during graph sampling. Node degree is used as an indicator of structural importance, allowing high-degree nodes to be preferentially retained in the sampled graph. Because such nodes often play important roles in graph connectivity, RD sampling is designed to preserve global topological characteristics. The sampling procedure is summarized as follows:

(1) Initialization

For the original graph $G\left(V,E\right)$, r nodes are randomly selected uniformly from the node set V as the initial node set. Here, $U\left(1,\left|V\right|\right)$ represents a uniform distribution within the interval $\left[1,\left|V\right|\right]$, where $\left|V\right|$ is the total number of nodes.

(1) $$\left\{Seeds\right\}=\left\{v_{i}|v_{i}\in V,i\sim U\left(1,\left|V\right|\right),\left|\left\{Seeds\right\}\right|=r\right\}$$

(2) Selecting nodes

For each current seed node $\left\{Seeds\right\}$, the set of its neighboring nodes is obtained and the degree of each neighboring node is calculated. The neighboring nodes are sorted in descending order based on $deg\left(u\right)$, and the top k neighboring nodes are selected from the sorted list; let $v_{1},v_{2},\cdots ,v_{k}$ be these neighboring nodes, and then add these neighboring nodes to the seed nodes:

(2) $$deg\left(u\right)=\left\{u|\left(u,v\right)\in E\right\}$$

(3) $$\left\{Seeds\right\}\leftarrow \left\{Seeds\right\}\cup \left\{v_{1},v_{2},\cdots ,v_{k}\right\}$$

(3) Subgraph construction

The above process is repeated until the number of seed nodes $\left\{Seeds\right\}\geq x$, where x represents the number of subgraph nodes to be sampled. The seed nodes are taken as the nodes of the subgraph $G_{RD}\left(V_{RD},E_{RD}\right)$, and the nodes in the subgraph are connected based on the edge connection relationships in the original graph.

The above procedure describes the original standalone RD algorithm. In the proposed RIGS framework, its degree-ranking principle is adapted to determine the next node at each hybrid sampling iteration, as described in Section Sampling Process.

### 3.2. Improved Forest Fire Sampling Algorithm Based on PageRank (IFFST-PR)

Improved Forest Fire Sampling based on PageRank (IFFST-PR) [6] combines community information with PageRank-based node importance to improve the preservation of the local community structure during graph sampling. Community coefficients are first used to identify candidate community centers, after which PageRank is employed to characterize their structural importance. Forest fire propagation is then performed within the identified communities to obtain the sampled nodes. The main steps are described below.

(1) Community coefficient

For a node n, the formula for calculating the community coefficient is as follows, where $d\left(n\right)$ represents the degree of node n, $n_{i},n_{j}$ represents the neighbors of node n, and $e\left(n_{i},n_{j}\right)=1$ indicates that two neighbors are connected.

(4) $$CC_{n}=\frac{2\sum_{i=1}^{d\left(n\right)}\sum_{j=1}^{d\left(n\right)}\left|e\left(n_{i},n_{j}\right)\right|}{d\left(n\right)\left(d\left(n\right)-1\right)}$$

(2) Choosing community center

If the $CC_{n}$ value of node n is greater than that of all its neighbors, then node n will be included in the community center set C.

(3) PageRank

The PageRank values of the nodes in the community center are calculated. Here, $PR\left(i\right)$ represents the PageRank value of node i, $N\left(i\right)$ is the set of neighbors of node i, $d\left(j\right)$ is the degree of node j, s is the damping coefficient, and n represents the number of nodes in the graph. Then, each community is sampled in sequence from the smallest to the largest PageRank value.

(5) $$PR\left(i\right)=\frac{1-s}{n}+s\sum_{j\in N\left(i\right)}\frac{PR\left(j\right)}{d\left(j\right)}$$

(4) Sampling

First, the sampling ratio for each community center is allocated. Here, i∈C denotes the community center set, and $r_{i}$ represents the sampling ratio. One node in each community is randomly selected as the ignition point and ignited. The spread starts from this ignition point and its neighboring nodes are ignited according to the fixed probability p. The burning is stopped when the number of sampling nodes in the community reaches the predetermined value.

(6) $$r_{i}=\frac{d\left(i\right)+\sum_{j\in N\left(i\right)}d\left(j\right)}{\sum_{k\in C}\left(d\left(k\right)+\sum_{m\in N\left(k\right)}d\left(m\right)\right)}$$

(5) Subgraph construction

After the sampling is completed, the obtained sampling nodes will be regarded as the nodes of the subgraph $G_{IFFST-PR}\left(V_{IFFST-PR},E_{IFFST-PR}\right)$, and the nodes in the subgraph will be connected according to the connection relationships of the edges in the original graph.

The complete procedure of the proposed IFFST-PR sampling algorithm is summarized in Algorithm 1.
**Algorithm 1** IFFST-PR Sampling Procedure1:**Input:** Graph
$G\left(V,E\right)$;
Fixed probability
p
2:**Output:** Sampled subgraph
$G_{IFFST-PR}\left(V_{IFFST-PR},E_{IFFST-PR}\right)$
3:Compute the community coefficient of each node using Equation (4)4:Select the community centers5:Compute PageRank value of each community center using Equation (5)6:Sort the communities according to their PageRank values7:Allocate the sampling budget for each community using Equation (6)8:**for** each community **do**9:Randomly select one ignition node10:
Activate neighboring nodes based on fixed probability
p
11:Repeat the spreading process until sampling budget is reached12:**end for**13:
Construct the sampled subgraph
$G_{IFFST-PR}$
14:
Return sampled subgraph
$G_{IFFST-PR}$


The above procedure describes the original standalone IFFST-PR algorithm. In RIGS, its PageRank-guided forest fire mechanism is adapted as a one-step node selection branch rather than being independently executed to generate a complete sampled subgraph, as described in Section Sampling Process.

### 3.3. GraphSAINT Normalization

GraphSAINT reduces the computational cost of large-scale GNN training by performing optimization on sampled subgraphs. However, because different nodes may have different probabilities of being included in the sampled subgraphs, directly computing the training objective on sampled nodes can introduce estimation bias. Consequently, the resulting subgraph-level objective may not accurately represent the contribution of nodes in the original graph.

To address this issue, GraphSAINT introduces a loss normalization mechanism [3]. Specifically, for each node v, its loss normalization coefficient is defined as $\lambda_{v}$, where $\left|V\right|$ is the total number of nodes in the graph and $p_{v}$ is the probability that node v is sampled.

In each subgraph training, only the sampled nodes are used to calculate the loss, and the above normalization coefficient is used to weight the node loss so as to ensure that the training loss of each subgraph is not biased, and the estimation bias caused by uneven sampling is eliminated.

(7) $$\lambda_{v}=\left|V\right|\cdot p_{v}$$

## 4. Proposed Methodology

This section presents the proposed RI Graph Sampling (RIGS) method in detail and describes its integration into a GCN-based botnet detection framework for large-scale communication graphs. The proposed framework is designed to alleviate the neighbor explosion problem while preserving informative graph structure during subgraph-based training.

### 4.1. RI Graph Sampling

The overall framework of RI Graph Sampling is illustrated in Figure 1. Unlike the conventional GraphSAINT sampling framework, which relies on individual stochastic sampling strategies, RIGS integrates the node selection principles of RD and IFFST-PR within a unified probabilistic sampling process.

As shown in Figure 1a, the method dynamically combines RD and IFFST-PR during subgraph construction. At each sampling step, the sampling strategy is selected probabilistically, where RD is chosen with probability 1−α and IFFST-PR is selected with probability α. The selected strategy determines the next sampled node and its associated edges, which are incrementally added into the sampled subgraph until the target number of sampled nodes is reached.

Figure 1b further illustrates the iterative hybrid sampling process. Starting from the original graph, RIGS probabilistically selects either the RD-based or the IFFST-PR-based node selection branch at each sampling step and progressively expands the sampled subgraph. Through this hybrid mechanism, RD contributes a preference for structurally important nodes associated with global topology, whereas IFFST-PR facilitates local community-oriented exploration. Their combination is intended to improve the preservation of both global and local structural information within the sampled subgraphs.

#### Sampling Process

In RI Graph Sampling, the sampled subgraph is constructed iteratively through a probabilistic hybrid sampling process. At each sampling step t, either the RD-based node selection branch or the IFFST-PR-based node selection branch is activated according to the hybrid sampling parameter α. Specifically, RD is selected with probability 1−α, whereas IFFST-PR is selected with probability α.

To describe this process more precisely, a random variable $u_{t}$ is generated from a uniform distribution $u_{t}\sim U\left(0,1\right)$.

The sampling branch at step t is then determined as follows:

(8) $$S_{t}=\left\{\begin{array}{l} RD,u_{t}<1-\alpha \\ IFFST-PR,u_{t}\geq 1-\alpha \end{array}\right.$$

where $S_{t}$ denotes the sampling branch selected at sampling step t.

It should be emphasized that RIGS does not independently execute the complete standalone RD or IFFST-PR algorithm at every sampling iteration. Instead, the node selection principles of the two methods are incorporated into two alternative branches that determine the next node to be added to the current sampled subgraph. This design enables RD and IFFST-PR to operate within the same iterative subgraph construction process.

When the RD branch is activated, the degree-ranking principle of RD is used to select the next node. Although the original standalone RD algorithm described in Section 3.1 may select multiple top- k neighboring nodes during a single expansion, RIGS applies this criterion in a step-wise manner. Specifically, only one candidate node is selected according to the degree-based ranking rule during each hybrid sampling iteration, and the selected node is then incorporated into the current sampled subgraph.

When the IFFST-PR branch is activated, RIGS performs a single PageRank-guided forest fire expansion from the current sampling frontier. Unlike the standalone IFFST-PR algorithm, which independently constructs a complete sampled subgraph, the IFFST-PR branch in RIGS performs only one node selection step. The next node is determined according to the forest fire exploration mechanism together with the precomputed PageRank information and is subsequently added to the current sampled subgraph.

Therefore, the hybridization of RD and IFFST-PR in RIGS occurs at the node selection level rather than at the complete-subgraph level. At each iteration, only one branch is activated, and the activated branch determines the next node to be incorporated into the sampled subgraph. The iterative process terminates when the number of sampled nodes reaches the predefined target size $N_{target}$.

The PageRank scores required by the IFFST-PR branch are computed once on the original graph before the hybrid sampling process begins and are subsequently reused throughout all sampling iterations. Consequently, PageRank calculation is treated as a one-time offline preprocessing operation rather than being repeatedly performed during online subgraph construction.

The complete RI Graph Sampling procedure is summarized in Algorithm 2.
**Algorithm 2** RI Graph Sampling Procedure1:**Input:** Botnet topology graph
$G\left(V,E\right)$;

Rank Degree *parameters* =
$\left(r,k,x\right)$;

IFFST-PR *parameters* =
$\left(p,s\right)$;

Hybrid sampling parameter
α;

Target sample size
$N_{target}$
2:**Output:** Sampled subgraph
$G_{RI}\left(V_{RI},E_{RI}\right)$
3:Initialize RD and IFFST-PR parameters4:
Compute PageRank scores on
$G\left(V,E\right)$
5:
Initialize sampled subgraph
$G_{RI}=\emptyset$
6:**while** $\left|V_{s}\right|<N_{target}$ **do**7:
Generate
$u\sim U\left(0,1\right)$
8:**if** u<1−α **then**9:Apply the RD-based node selection rule to obtain the next sampled node10:**else**11:Apply one-step IFFST-PR-based node selection to obtain the next sampled node12:**end if**13:
Add sampled node and associated edges into
$G_{RI}$
14:**end while**15:
Return sampled subgraph
$G_{RI}\left(V_{RI},E_{RI}\right)$


### 4.2. Botnet Detection Process Based on RI Graph Sampling

This section describes how RI Graph Sampling is integrated into the proposed botnet detection framework. As illustrated in Figure 2, the overall detection procedure first transforms raw network traffic into a communication graph, after which the training nodes are sampled using RIGS and processed by a GCN for node classification. The framework is subsequently evaluated in terms of its computational complexity. By restricting GNN computation to structurally informative sampled subgraphs, the proposed framework is intended to improve training efficiency on large-scale botnet graphs while retaining information required for node classification.

#### 4.2.1. Graph Construction

The graph construction module transforms raw network flow records into a graph-structured representation suitable for subsequent sampling and GNN training. As illustrated in Figure 3, each network flow record describes communication between a source IP address (SrcIP) and a destination IP address (DstIP), together with traffic statistics such as flow duration (Dur), transmitted bytes (SrcBytes), and received bytes (DstBytes).

The graph construction process consists of two main stages: graph transformation [28] and node feature extraction. During graph transformation, host IP addresses are represented as graph nodes, and each communication flow from a source IP to a destination IP is represented as a directed edge. This process converts individual flow records into a host-level communication topology that captures the interaction relationships among network entities.

During node feature extraction, multiple flows associated with the same IP node are statistically aggregated to obtain behavioral features. Specifically, five node features—Conn, FailConn, Dur, SrcBytes, and DstBytes—are extracted to characterize connection behavior, flow duration, and traffic volume. These features form the node feature matrix used as input to the subsequent GCN model. The detailed definitions and calculation procedures are provided in Section 5.3.2.

As shown in Figure 3, the graph construction module first maps each host IP in the raw network flow file to a node in the graph and models the communication from the source IP to the destination IP as a directed edge, thereby forming a complete host communication topology. On this basis, multiple network flows associated with the same node are statistically aggregated to extract five features: successful connections, failed connections, flow duration, and transmitted and received bytes. Through this process, the raw network flow data are ultimately converted into graph-structured data, providing structured and information-rich input for subsequent graph sampling and model training.

#### 4.2.2. GCN Model Construction

The Graph Convolutional Network (GCN) is a type of Graph Neural Network (GNN) model. GCN learns representations from graph-structured data by aggregating information from a node and its neighboring nodes. Its input consists of the node feature matrix and the adjacency matrix. The propagation rule of GCN is defined as follows:

(9) $$X^{\left(l+1\right)}=\sigma \left({\tilde{D}}^{-\frac{1}{2}}\tilde{A}{\tilde{D}}^{-\frac{1}{2}}X^{\left(l\right)}W^{\left(l\right)}\right)$$

where $X^{\left(l\right)}$ denotes the node feature matrix of the l-th layer; $\tilde{A}=A+I$ represents the adjacency matrix with added self-loops; $\tilde{D}$ is the degree matrix of $\tilde{A}$; ${\tilde{D}}^{-\frac{1}{2}}\tilde{A}{\tilde{D}}^{-\frac{1}{2}}$ denotes the symmetric normalization of the $\tilde{A}$; σ represents the ReLU activation function; and $W^{\left(l\right)}$ is the weight matrix corresponding to the features of the previous layer.

#### 4.2.3. Algorithm Complexity Analysis

To evaluate the computational efficiency of the proposed RI Graph Sampling for large-scale botnet detection, the overall computational complexity is analyzed by separately considering the one-time offline preprocessing stage and the online hybrid sampling stage.

Let the original graph be $G=\left(V,E\right)$, where $\left|V\right|=N$ and $\left|E\right|=M$. Assume that the target sampled subgraph contains $n_{s}$ sampled nodes. In RI Graph Sampling, the sampled subgraph is constructed iteratively through a hybrid sampling mechanism. At each sampling step, RD is selected with probability 1−α, while IFFST-PR is selected with probability α.

For the RD sampling strategy, the algorithm accesses the neighboring nodes of the current sampled node and ranks them according to node degree. Let the average degree of the graph be d. The computational complexity of processing one sampled node is approximately $O\left(d\log d\right)$.

Therefore, for $n_{s}$ sampled nodes, the overall time complexity of the RD component can be approximated as $O\left(n_{s}d\log d\right)$.

For the IFFST-PR sampling strategy, the computational overhead consists of two components: a one-time offline preprocessing stage and an online sampling stage. During preprocessing, the PageRank scores of the original graph are computed once to identify community-aware sampling priorities. Assuming that the PageRank algorithm performs k iterations on the original graph containing M edges, the preprocessing complexity is $O\left(kM\right)$.

During online sampling, the precomputed PageRank scores are directly reused, and no additional PageRank computation is required. Consequently, the additional computational cost is confined to the offline preprocessing stage and does not affect the per-iteration online sampling complexity. Let $m_{s}$ denote the number of edges in the sampled subgraph. The corresponding online sampling complexity is $O\left(m_{s}\right)$.

Since RI Graph Sampling probabilistically selects the sampling strategy at each iteration, the expected time complexity of the hybrid sampling process can be expressed as $O\left(\left(1-\alpha \right)n_{s}d\log d+\alpha m_{s}\right)$.

Since the PageRank scores are precomputed during the offline preprocessing stage, the PageRank computation cost $O\left(kM\right)$ is excluded from the online sampling complexity. Therefore, only the costs of RD sampling and IFFST-PR graph traversal are considered during sampling.

Assuming that the GCN contains a fixed number of layers, the per-epoch message passing complexity is approximately $O\left(MF\right)$ per layer for each training epoch, where F is the feature dimension, RI Graph Sampling performs graph convolution only on sampled subgraphs with $m_{s}\ll M$.

Compared with the original GraphSAINT, RI Graph Sampling introduces only an additional one-time preprocessing cost $O\left(kM\right)$, while preserving the same online sampling complexity.

## 5. Experiment and Analysis

This section evaluates the proposed RI Graph Sampling-based botnet detection framework. We first introduce the datasets and evaluation metrics used in the experiments, followed by the data preprocessing, node feature extraction, sampling normalization, and experimental configuration. We then evaluate the proposed method in terms of detection performance, computational efficiency, parameter sensitivity, ablation results, and structural preservation. The experimental environment is summarized in Table 1.

### 5.1. Dataset

The experiments are conducted on two publicly available botnet datasets, CTU-13 and NCC-2. The CTU-13 dataset contains 13 botnet scenarios, with the traffic statistics of each scenario summarized in Table 2.

The NCC-2 dataset contains traffic collected from three network sensors and represents scenarios in which multiple botnets interact with or attack different monitored networks. The dataset provides three individual sensor scenarios and an additional merged scenario that combines data from all three sensors. The corresponding statistics are summarized in Table 3.

#### 5.1.1. Graph Transformation

Each CTU-13 and NCC-2 scenario is independently transformed into a communication graph. Specifically, each host IP address is represented as a node, and each network flow from a source IP address to a destination IP address is represented as a directed edge. The resulting graph therefore captures host-level communication relationships within each scenario. The graph statistics of the CTU-13 and NCC-2 datasets are summarized in Table 4 and Table 5, respectively.

#### 5.1.2. Selection of Experimental Datasets for Large-Scale Graph Node Classification

After graph construction, four representative CTU-13 scenarios together with the merged NCC-2 graph are selected for subsequent experiments. Because GraphSAINT [3], ClusterGCN [18], and SHADOW-GCN [29] are primarily designed for large-scale graph learning, CTU-13 scenarios with relatively large communication graphs are prioritized. Specifically, Scenario 9 (Neris), Scenario 10 (Rbot_1), Scenario 11 (Rbot_2), and Scenario 12 (NSIS.ay) are selected. Each scenario is independently represented as a communication graph without artificially merging unrelated CTU-13 scenarios, thereby preserving its original communication topology. The statistics of the five experimental graphs are summarized in Table 6.

Benign nodes are partitioned into training, validation, and test subsets at an 8:1:1 ratio. Because the number of botnet nodes is extremely limited, botnet nodes are assigned separately to ensure that each subset contains positive samples whenever possible. In particular, Rbot_2 and NSIS.ay each contain only three botnet nodes; therefore, one botnet node is assigned to each of the training, validation, and test subsets.

### 5.2. Evaluation Metrics

The evaluation metrics used in this study include accuracy (ACC), macro-averaged precision (Macro-Precision), macro-averaged recall (Macro-Recall), macro-averaged F1-score (Macro-F1), Matthews correlation coefficient (MCC), and runtime. Accuracy measures the proportion of correctly classified nodes among all evaluated nodes. Considering the severe class imbalance between benign and botnet nodes, Precision, Recall, and F1-score are reported using macro averaging. Specifically, each metric is first calculated independently for the benign and botnet classes, and the arithmetic mean of the class-wise scores is then reported. Consequently, both classes contribute equally to the final metric regardless of their population sizes. MCC is additionally adopted because it jointly considers true positives, true negatives, false positives, and false negatives and is therefore suitable for evaluating classification performance under highly imbalanced class distributions. The calculation formulas for accuracy, macro-averaged precision, macro-averaged recall, macro-averaged F1-score, and MCC are as follows:

(10) $$Accuracy=\frac{TP+TN}{TP+TN+FP+FN}$$

(11) $$Precision_{macro}=\frac{1}{C}\sum_{c=1}^{C}\frac{TP_{c}}{TP_{c}+FP_{c}}$$

(12) Recallmacro=1C∑c=1CTPcTPc+FNc

(13) $$F1_{macro}=\frac{1}{C}\sum_{c=1}^{C}\frac{2\times TP_{c}}{2\times TP_{c}+FP_{c}+FN_{c}}$$

(14) $$MCC=\frac{TP\times TN-FP\times FN}{\sqrt{\left(TP+FP\right)\left(TP+FN\right)\left(TN+FP\right)\left(TN+FN\right)}}$$

where TP, TN, FP, and FN denote the numbers of true positives, true negatives, false positives, and false negatives, respectively, in the binary confusion matrix. For the macro-averaged metrics, $TP_{c}$, $FP_{c}$ and $FN_{c}$ denote the class-specific true-positive, false-positive, and false-negative counts obtained by treating class c as the positive class in a one-vs-rest manner. C denotes the total number of classes. In this study, C=2, corresponding to the benign and botnet classes. The class-wise Precision, Recall, and F1-score are calculated independently for each class and then averaged with equal weight to obtain the corresponding macro-averaged metrics.

For binary node classification, no additional threshold optimization is performed. The predicted label of node i is determined according to the class with the maximum output logit:

(15) $${\hat{y}}_{i}=\arg\underset{c\in \{0,1\}}{\max}z_{i,c}$$

where $z_{i,c}$ denotes the output logit of node i for class c. In the binary classification setting, this decision rule is equivalent to applying a probability threshold of 0.5 to the positive-class probability obtained after the Softmax operation. The same decision rule is consistently adopted across all datasets and comparison methods.

### 5.3. Data Preprocessing and Feature Selection

Data preprocessing and feature selection are important steps in transforming network flows into graph-structured data, enabling subsequent sampling and learning on the dataset. In this section, we describe in detail the processes of data preprocessing and node feature extraction. These steps not only help clean the raw data but also effectively reduce data noise, thereby improving the accuracy and generalization ability of GNN in botnet detection tasks.

#### 5.3.1. Data Preprocessing

Before graph construction, the raw traffic records are processed using a unified preprocessing procedure consisting of four steps: data splitting, data cleaning, feature encoding, and label assignment.

(1) Data Splitting

Following the node classification setting adopted by GraphSAINT, each dataset is represented as a single communication graph $G\left(V,E\right)$, where each host IP address corresponds to a unique node and each network flow is represented as a directed edge between the source and destination IP addresses. The nodes are partitioned into training, validation, and test subsets using three mutually exclusive masks, denoted as train mask, validation mask, and test mask, with an 8:1:1 ratio (see Table 7).

This study adopts a transductive node-classification setting. Each dataset is represented as a single communication graph $G\left(V,E\right)$, and the nodes are partitioned into training, validation, and test subsets using three mutually exclusive masks with an 8:1:1 ratio. During training, the graph topology and node features of all nodes, including validation and test nodes, remain available to the GCN and may contribute to message passing when they are included in the sampled subgraphs. In particular, edges incident to validation or test nodes are not removed from the communication graph. However, supervision is strictly restricted to the training nodes: only the labels and losses of nodes indicated by the training mask are used for parameter optimization. The labels of validation and test nodes are not accessed during training and do not contribute to gradient computation or model parameter updates. The validation mask is used only for model selection, whereas the test mask is used exclusively for final performance evaluation. Therefore, validation and test nodes may provide unlabeled structural and feature information through message passing, but no validation or test label information is used during model optimization.

(2) Data Cleaning

We remove records with missing values, infinite values and duplicate entries in the raw traffic data so as to avoid adverse effects on model training.

(3) Feature Encoding

Non-numeric fields such as IP addresses and label identifiers are converted into numerical representations to adapt to the input requirements of the GNN model.

(4) Label Assignment

The CTU-13 and NCC-2 datasets provide ground-truth labels for all nodes. During graph construction, these labels are directly converted into binary class labels, where botnet nodes are represented by 1 (positive class) and normal nodes are represented by 0 (negative class). This binary labeling strategy is consistently adopted for all datasets used in the experiments.

#### 5.3.2. Node Feature Extraction

Following the node feature design specified in Section 4.2.1, node features are aggregated at the IP-address level. Conn, FailConn, Dur, and SrcBytes characterize the outgoing communication behavior of each node and are aggregated according to the source IP, whereas DstBytes characterizes the incoming traffic behavior and is aggregated according to the destination IP. The detailed definitions and aggregation rules of the five node features are summarized in Table 8.

For Dur, flow records with zero duration are excluded from the averaging operation because they correspond to failed or incomplete connections. SrcBytes is calculated by averaging the transmitted bytes of the flow records associated with each source IP. DstBytes is obtained from the received traffic volume of each flow and then averaged according to the destination IP, thereby characterizing the incoming traffic behavior of a node when acting as a receiver. The resulting five-dimensional feature vector is assigned to each IP node and used as the input node representation for subsequent GNN training.

#### 5.3.3. Sampling Normalization

Since different nodes may have different probabilities of being sampled during the hybrid sampling process, RI Graph Sampling adopts the sampling normalization mechanism proposed in GraphSAINT to reduce the estimation bias caused by unequal sampling probabilities.

Assume that the probability of node $v_{i}$ being sampled under RD and IFFST-PR are $p_{i}^{\left(RD\right)}$ and $p_{i}^{\left(IFFST-PR\right)}$, respectively. During the hybrid sampling process, the final probability that node i is sampled can be defined as follows:

(16) $${\hat{p}}_{i}=\frac{1}{R}\sum_{r=1}^{R}I\left(i\in S^{\left(r\right)}\right)$$

where R denotes the number of independent sampling repetitions, $S^{\left(r\right)}$ denotes the sampled node set obtained in the r-th RIGS execution, and $I\left(\cdot \right)$ is the indicator function. The estimated marginal inclusion probability ${\hat{p}}_{i}$ is then used to compute the sampling normalization coefficient.

Based on the normalization strategy of GraphSAINT, the normalization coefficient of node $v_{i}$ is defined as follows:

(17) $$\lambda_{i}=\left|V\right|\cdot {\hat{p}}_{i}$$

where $\left|V\right|$ denotes the number of nodes in the original graph and ${\hat{p}}_{i}$ represents the probability that node $v_{i}$ is sampled. In our implementation, R is set to 30. Before model training, RIGS is independently executed 30 times for each dataset, and the number of times each node appears in the sampled subgraphs is recorded. The marginal inclusion probability of each node is estimated as its occurrence frequency over these 30 sampling runs. The estimated probabilities are then used to compute the GraphSAINT normalization coefficients according to Equation (17). This estimation is performed once for each dataset as an offline preprocessing step, and the resulting normalization coefficients are subsequently reused throughout model training rather than being re-estimated at every epoch.

Through the normalization mechanism, RI Graph Sampling effectively reduces the estimation bias introduced by stochastic hybrid sampling and improves the stability of GNN training.

#### 5.3.4. Loss Function

Due to the severe class imbalance between benign and botnet nodes, Focal Loss is employed as the training objective instead of conventional cross-entropy loss. Focal Loss reduces the contribution of easily classified majority-class samples and increases the relative importance of difficult samples during optimization, thereby alleviating the bias toward benign nodes without changing the original graph structure or class distribution.

(18) $$L_{FL}=-\alpha_{t}{\left(1-p_{t}\right)}^{\gamma }\log\left(p_{t}\right)$$

where $p_{t}$ denotes the predicted probability of the ground-truth class, $\alpha_{t}$ is the class-balancing factor, and γ is the focusing parameter that controls the reduction in loss contribution from easily classified samples.

#### 5.3.5. Experimental Configuration

To facilitate reproducibility, the main experimental parameters are summarized in Table 9. Unless otherwise stated, the same settings are used throughout the experiments. The target subgraph size is 6000, and the hybrid coefficient is set to 0.6 for RD and 0.4 for IFFST-PR. In the RD component, neighboring nodes are ranked by degree during sampling. In the IFFST-PR component, the forest fire propagation probability is 0.4, while PageRank uses damping coefficient 0.85 and a maximum of 100 iterations. PageRank is computed once during offline preprocessing.

The detection model employs a two-layer GCN with hidden dimension 256, a learning rate of 0.001, Adam optimizer, and dropout rate 0.2. The model is trained for 50 epochs, with 50 sampled subgraphs generated per epoch. Each experiment is independently repeated 5 times using predefined random seeds {42, 43, 44, 45, 46}, and the results are reported as mean± standard deviation. The focusing parameter is set to γ=2, and the balancing factor is set to $\alpha_{t}=0.75$ in all experiments.

The complete configuration used in the experiments is summarized in Table 9.

### 5.4. Experimental Procedure and Results Analysis

#### 5.4.1. RI Graph Sampling Training

The RIGS-based detection model is trained using the configuration summarized in Table 9. The GCN contains two layers and is trained for 50 epochs with a learning rate of 0.001. During each epoch, RIGS generates sampled subgraphs to restrict the computational scope of GCN training.

Figure 4 shows the training-loss convergence curve of the RIGS-based model. The loss is relatively high and exhibits noticeable fluctuations during the early training stage because stochastic subgraph sampling produces different node and edge compositions across training batches. As training progresses, the loss shows an overall downward trend and gradually stabilizes, indicating convergence of the optimization process.

#### 5.4.2. Computational Scalability Evaluation on Different Graph Sizes

To evaluate the computational scalability of RI Graph Sampling on large-scale graph data, experiments were conducted on progressively larger graph subsets constructed from the NCC-2 dataset. Specifically, four graph scales containing approximately 100 K, 300 K, 700 K, and 1.4 M nodes were considered. To ensure that each graph subset contained sufficient positive samples for the botnet detection task, all 17 botnet nodes were retained, while normal nodes were randomly sampled to obtain the target graph size.

Since the number of botnet nodes remains fixed, while the number of normal nodes increases, the class prevalence varies across graph scales, as reported in Table 10. This experiment focuses exclusively on computational scalability, evaluated in terms of training time and peak GPU memory consumption.

Figure 5 compares GraphSAGE, GraphSAINT-RW, and RI Graph Sampling under the four graph scales. As shown in Figure 5a, the training time of all methods increases with graph size. GraphSAGE exhibits the most pronounced growth in training time, whereas the two subgraph-based approaches show slower increases. RI Graph Sampling requires less training time across the evaluated graph sizes, indicating favorable computational scalability as the graph size increases.

Figure 5b presents the corresponding peak GPU memory consumption. The memory usage of all methods increases as the graph becomes larger, while RI Graph Sampling maintains lower peak GPU memory consumption than the compared methods. This result indicates that restricting GNN computation to structurally informative sampled subgraphs can reduce the memory overhead associated with large-scale graph learning.

Overall, the results demonstrate that RI Graph Sampling provides favorable computational scalability in terms of both training time and GPU memory consumption.

#### 5.4.3. Performance Comparison of RI Graph Sampling with Baseline Methods

To systematically evaluate the performance of RI Graph Sampling, we select seven representative methods as baselines for comparison.

GCN [30]: a classical graph convolutional network that performs message passing over the entire graph. It serves as the full-graph training baseline but suffers from severe neighbor explosion and high computational cost on large-scale graphs.

GraphSAGE [14]: a representative node-wise sampling method that randomly samples a fixed number of neighbors for each node at every layer, effectively limiting the aggregation scope.

ClusterGCN [18]: a clustering-based subgraph sampling method. It partitions the original graph into multiple clusters via graph clustering, and randomly selects clusters as training subgraphs to reduce computational overhead.

LMC [20]: a subgraph-based GNN training method that compensates for messages from out-of-batch nodes to improve gradient estimation during mini-batch training. It enhances the accuracy and convergence of subgraph sampling while maintaining computational efficiency.

GraphSAINT-Node/Edge/RW [3]: three sampling strategies in the GraphSAINT framework, based on random node sampling, random edge sampling, and random walk sampling, respectively. These methods serve as the primary baselines for evaluating the effectiveness of the proposed hybrid sampling strategy.

SHADOW-GCN [29]: a decoupled graph neural network framework. It separates the depth of GNN from the scope of receptive field, and improves training efficiency on large-scale graphs through hidden subgraph construction.

All baseline methods use the same node features, train/validation/test partitions, hardware environment, and evaluation protocol. Unless required by the corresponding architecture, common training settings, including 50 epochs and the same predefined random seeds, are kept consistent. Method-specific hyperparameters, such as neighbor-sampling fan-outs, graph-partition settings, subgraph budgets, and random-walk parameters, follow the recommended configurations of the corresponding implementations under comparable tuning budgets. The settings used in our experiments are summarized in Table 11.

Table 12, Table 13, Table 14, Table 15 and Table 16 present the ACC, Precision, Recall, F1-score and MCC of all methods on five botnet datasets.

Table 12, Table 13, Table 14, Table 15 and Table 16 present the ACC, Precision, Recall, F1-score, and MCC results of all compared methods across the five botnet datasets. Overall, RI Graph Sampling maintains competitive detection performance across different datasets, although it does not consistently achieve the best result for every evaluation metric. Several baseline methods obtain higher Precision, Recall, or F1-score on individual datasets.

It should be noted that the dataset-level classification results must be interpreted with caution because the positive test classes are extremely small. As reported in Table 7, the test subsets of Neris, Rbot_1, Rbot_2, and NSIS.ay each contain only one botnet node, while the NCC-2 test subset contains only two botnet nodes. Consequently, the classification outcome of a single positive node can substantially affect minority-class Recall, Macro-F1, MCC, and related metrics. Therefore, small numerical differences between methods on an individual dataset should not be interpreted as strong evidence of classification superiority. The dataset-level results are instead used to characterize the overall performance tendencies of the compared methods under the highly imbalanced botnet-detection setting.

Compared with GCN, GraphSAGE, ClusterGCN, LMC, SHADOW-GCN, and the GraphSAINT sampling variants, RIGS does not exhibit a consistent advantage in classification performance alone. Nevertheless, the results show that the proposed hybrid sampling strategy retains sufficient discriminative information for botnet detection while substantially reducing the computational scope of GNN training. Therefore, the primary advantage of RIGS lies in achieving competitive classification performance together with improved computational efficiency and structural preservation.

This observation suggests that RIGS should not be interpreted as a method designed to maximize detection accuracy on every dataset. Instead, it provides a favorable trade-off among classification performance, computational efficiency, and structural preservation, which is particularly important for large-scale botnet detection scenarios.

Table 17 presents the end-to-end computational cost of different methods on the NCC-2 dataset, including preprocessing time, sampling time, training time, total time, and peak GPU memory consumption. In this study, preprocessing time refers to the one-time method-specific operations performed before iterative subgraph sampling and model training, whereas sampling time denotes the computational time required to generate or select the training subgraphs after preprocessing and excludes GNN forward propagation, backpropagation, and parameter updates. Training time refers to the time spent on model forward propagation, loss computation, backpropagation, and parameter optimization. The total time is calculated as the sum of preprocessing, sampling, and training time.

For RI Graph Sampling, the preprocessing stage mainly consists of the one-time PageRank computation required by IFFST-PR, while the sampling stage corresponds to the subsequent hybrid subgraph construction using the RD-based and IFFST-PR-based node selection branches. The PageRank scores are computed only once and reused throughout all subsequent sampling iterations. Therefore, although RIGS introduces a preprocessing cost of 7.8 ± 0.3 s, this cost does not recur during iterative sampling or model training.

Overall, RI Graph Sampling achieves the best computational efficiency among all compared methods. Specifically, RIGS obtains the lowest training time of 27.2 ± 0.7 s and the lowest total wall-clock time of 40.9 ± 0.8 s. Even when the one-time PageRank preprocessing cost is fully included, its total execution time remains lower than that of GraphSAINT-RW and substantially lower than those of the other baseline methods. This demonstrates that the additional PageRank preprocessing overhead is effectively compensated for by the improved efficiency of the subsequent sampling and training stages.

In addition, RIGS requires only 3.26 ± 0.09 GB of peak GPU memory, which is the lowest among all evaluated methods. Compared with full-graph GCN and other sampling-based approaches, the proposed method substantially reduces memory consumption while maintaining the shortest overall execution time. These results demonstrate that RI Graph Sampling achieves the best overall trade-off in computational cost and memory efficiency, confirming its effectiveness and scalability for large-scale botnet detection.

Figure 6 plots the F1-score convergence curves over training time on the NCC-2 dataset, the largest graph in our experiments. As shown in the figure, RI Graph Sampling converges within a shorter training time than all baseline methods: it reaches a competitive F1-score level in much less training time and stabilizes at its final performance earlier. This convergence pattern is consistently observed across all four CTU-13 datasets.

This result confirms that the proposed hybrid sampling strategy effectively reduces per-iteration training cost while preserving detection performance, delivering higher overall training efficiency for large-scale botnet detection.

#### 5.4.4. Sensitivity Analysis of the Hybrid Sampling Parameter

To investigate the sensitivity of RI Graph Sampling to the hybrid sampling parameter α, we varied α from 0 to 1 with an interval of 0.2 while keeping all other experimental settings unchanged. Here, 1−α denotes the probability of selecting RD sampling, whereas α represents the probability of selecting IFFST-PR sampling. The experimental results are shown in Figure 7.

As shown in Figure 7, a similar trend is observed across all five datasets. As α increases from 0 to 0.4, the Accuracy, Recall, and F1-score generally improve, indicating that combining the RD-based global structural preference with the IFFST-PR-based local community exploration provides complementary structural information. When α is further increased beyond 0.4, the overall detection performance gradually decreases or remains close to its peak value.

Among the evaluated parameter settings, α=0.4 provides near-best overall performance across the five datasets. In particular, the performance at α=0.4 is competitive for all three evaluation metrics Therefore, α=0.4 is adopted as the default hybrid sampling parameter in the subsequent experiments.

#### 5.4.5. Scalability Comparison Between GraphSAGE and GraphSAINT Frameworks

To examine the scalability of the GraphSAINT sampling framework across different GNN architectures, GraphSAGE and GraphSAINT are evaluated with three representative models: GCN, GAT [31], and JK-Net [32]. For each architecture, both two-layer and four-layer configurations are considered, and training time and validation F1-score are compared.

Figure 8 illustrates the variations in training time and validation F1-score for the GraphSAGE and GraphSAINT sampling frameworks under different model architectures and layer configurations. It can be observed that, across all three GNN models, the GraphSAINT framework consistently demonstrates faster convergence under the same network depth and is able to achieve higher validation F1-scores within a shorter training time. This phenomenon is particularly evident in the GAT and JK-Net models, indicating that GraphSAINT has a clear advantage in training efficiency when handling graph neural networks with more complex structures and larger numbers of parameters.

A further comparison between models with different depths shows that when the network depth increases from 2 layers to 4 layers, the GraphSAGE sampling framework exhibits noticeable performance degradation and training instability. The validation F1-score fluctuates significantly, and the training time increases substantially. This is mainly because GraphSAGE adopts a neighbor expansion–based sampling strategy, which is prone to the “neighbor explosion” problem in deeper networks. As a result, the computational graph grows rapidly, adversely affecting both training efficiency and model stability.

In contrast, GraphSAINT performs random subgraph sampling and applies normalization at the subgraph level, effectively limiting the number of nodes and edges involved in each training iteration. As a result, even when the network depth increases, the model can maintain a relatively stable training process and high validation performance. These results indicate that the GraphSAINT sampling strategy possesses strong generalization ability and scalability across different graph neural network architectures.

#### 5.4.6. Ablation Analysis of RD and IFFST-PR

To evaluate the contribution of the two sampling components, we compare standalone RD sampling, standalone IFFST-PR sampling, and the proposed hybrid RIGS method under the same experimental setting. The results are reported in Table 18, Table 19 and Table 20.

As shown in Table 18, Table 19 and Table 20, the proposed RI Graph Sampling achieves competitive overall performance across the five datasets. In particular, the hybrid strategy obtains clear improvements in several datasets, such as Neris and Rbot_2, while maintaining comparable performance on the remaining datasets. However, the individual RD or IFFST-PR strategies achieve better results for certain metrics, such as Recall and F1-score on Rbot_1 and NCC-2. This indicates that the relative effectiveness of the two sampling components varies with the structural characteristics of different network graphs. Overall, the results suggest that combining RD and IFFST-PR provides complementary structural information and enables RI Graph Sampling to achieve a more balanced performance across different datasets, rather than relying solely on either global topology-oriented or local community-oriented sampling.

#### 5.4.7. Ablation Experiments of Flow Feature and Topology Feature

In the RI Graph Sampling botnet detection system, detection performance comes from two complementary information sources: flow features and topological features. To evaluate the contribution of each part and verify the effectiveness of feature-structure fusion, we conduct ablation experiments under three experimental configurations, defined as follows:

1. Flow: Only the five manually aggregated traffic statistical features (Conn, FailConn, Dur, SrcBytes, DstBytes) are used as node input, and classification is performed with a fully connected network without GCN. This configuration completely excludes graph topology information and only relies on traffic behavior features for detection.
2. Topology: Only the adjacency structure of the communication graph is used for GCN learning, and no handcrafted traffic statistical features are introduced. In this setup, the model learns node representations purely from topological connectivity patterns, and the discriminative information comes entirely from implicit graph structural features extracted by GCN.
3. Flow + Topology: The five traffic features are used as initial node attributes, and node representations are learned through GCN. In this configuration, the model simultaneously captures traffic behavior attributes and graph topological structure information, which is the complete setup of our proposed method.

The experimental results are shown in Figure 9.

As shown in Figure 9, both network flow features and topological features contribute to botnet detection, while their combination consistently achieves the highly competitive ACC, Recall, and F1-score across the five datasets. These results indicate that network flow features and topological features provide complementary information, and their integration enables richer graph representations, thereby improving the effectiveness and robustness of RI Graph Sampling for large-scale botnet detection.

#### 5.4.8. Structural Preservation Analysis of Sampled Subgraphs

To provide a more comprehensive evaluation of structural preservation, six metrics are considered on the NCC-2 dataset: average degree, Kolmogorov–Smirnov (KS) distance, largest connected component (LCC) ratio, average clustering coefficient, normalized mutual information (NMI), and community coverage. The original full graph is used as the structural reference. Average degree is reported as an auxiliary indicator of whether a sampling method preferentially retains highly connected nodes.

Average degree is retained as an auxiliary indicator to characterize the tendency of a sampling method to preserve highly connected nodes.

The KS distance is used to quantify the difference between the degree distributions of the original graph and the sampled subgraph. It is defined as follows:

(19) $$D_{KS}\left(G,Gs\right)=\underset{k}{sup}\left|F_{G}\left(k\right)-F_{G_{s}}\left(k\right)\right|$$

where $F_{G}\left(k\right)$ and $F_{G_{s}}\left(k\right)$ denote the cumulative distribution functions of node degrees in the original graph G and sampled subgraph $G_{s}$, respectively. A smaller KS distance indicates a higher similarity between the two degree distributions, with $D_{KS}=0$ representing identical distributions.

The LCC ratio measures the connectivity retained in the sampled subgraph and is defined as follows:

(20) $$R_{\mathrm{LCC}}\left(G_{s}\right)=\frac{|V\left(C_{\max}\left(G_{s}\right)\right)|}{|V_{s}|}$$

where $C_{\max}\left(G_{s}\right)$ denotes the largest connected component of $G_{s}$, $V\left(C_{\max}\left(G_{s}\right)\right)$ is its node set, and $|V_{s}|$ is the total number of nodes in the sampled subgraph. A value closer to the LCC ratio of the original graph indicates better preservation of global graph connectivity.

The average clustering coefficient (CC) [33] measures the preservation of local connectivity patterns, defined in Equation (4).

NMI is employed to evaluate the consistency of community assignments between the original graph and the sampled subgraph. Let $C_{G}\left(V_{s}\right)$ denote the community labels of the sampled nodes $V_{s}$ inherited from the original graph, and let $C_{G_{s}}$ denote the community labels obtained by applying the same community detection algorithm to the sampled subgraph. NMI is defined as follows:

(21) $$\mathrm{NMI}\left(G,\;G_{s}\right)=\frac{2I\left(C_{G};\;\left(V_{s}\right)C_{G_{s}}\right)}{H\left(C_{G}\left(V_{s}\right)\right)+H\left(C_{G_{s}}\right)}$$

where $I\left(\cdot ;\;\cdot \right)$ denotes mutual information and $H\left(\cdot \right)$ denotes entropy. NMI ranges from 0 to 1, and a larger value indicates greater consistency between the community structures of the original and sampled graphs.

Community coverage measures the proportion of communities in the original graph that are represented by at least one node in the sampled subgraph. Let the community set of the original graph be $C_{G}=\left\{C_{1},\;C_{2},\;\ldots ,\;C_{M}\right\}$.

The community coverage is defined as follows:

(22) $$R_{cov}\left(G_{s}\right)=\frac{\left|\left\{C_{j}\in C_{G}|C_{j}\cap V_{s}\neq \emptyset \right\}\right|}{\left|C_{G}\right|}$$

A larger coverage value indicates that the sampled subgraph represents a broader range of communities in the original graph, with a value of 1 indicating that all original communities are covered.

The experimental results are presented in Table 21. The results demonstrate that different sampling strategies exhibit distinct structural preservation characteristics. GraphSAINT-Node, GraphSAINT-Edge, and GraphSAINT-RW maintain high LCC ratios, indicating that the overall connectivity of the sampled graphs is preserved. However, their low clustering coefficients and NMI values indicate limited capability in preserving local connectivity patterns and community structures.

RD Graph Sampling strongly favors highly connected nodes, resulting in an average degree of 16.3925 ± 0.6247, which is substantially higher than that of the original graph. Although RD maintains a high LCC ratio of 0.9992 ± 0.0001, its KS distance of 0.3357 ± 0.0026 indicates a considerable deviation from the original degree distribution. In contrast, IFFST-PR Graph Sampling achieves an average clustering coefficient of 0.0428 ± 0.0002, which is the closest to the original value of 0.0482, and obtains an NMI of 0.7215 ± 0.0017, demonstrating its stronger capability in preserving local community structure. However, its LCC ratio decreases to 0.8602 ± 0.0018, indicating a certain loss of global connectivity.

RI Graph Sampling provides a more balanced preservation of both global and local structures. In particular, it achieves the lowest KS distance of 0.0672 ± 0.0004, indicating that its degree distribution is substantially closer to that of the original graph than those of the other sampling methods. Meanwhile, its LCC ratio remains high at 0.9990 ± 0.0001, showing that the global connectivity of the original graph is effectively retained. For local and community structures, RI Graph Sampling achieves an average clustering coefficient of 0.0352 ± 0.0002, an NMI of 0.8124 ± 0.0012, and a community coverage of 0.6909 ± 0.0011. Among all sampling methods, RIGS obtains the highest NMI and community coverage, while maintaining a clustering coefficient relatively close to that of the original graph.

Overall, the results indicate that RD primarily preserves highly connected nodes and global connectivity, whereas IFFST-PR exhibits stronger preservation of local clustering characteristics. By integrating the complementary properties of these two strategies, RI Graph Sampling achieves the best degree-distribution similarity while simultaneously maintaining high graph connectivity and superior community consistency and coverage. These results provide broader structural evidence that the proposed hybrid sampling strategy achieves an effective balance between global topology preservation and local community structure preservation.

## 6. Conclusions and Future Work

This study proposes RI Graph Sampling (RIGS), a hybrid graph sampling method for large-scale botnet detection. RIGS probabilistically integrates an RD-based node selection branch with an IFFST-PR-based node selection branch to combine their complementary preferences for global topology and local community structure. The proposed sampler is incorporated into a GraphSAINT-based training framework with sampling normalization and GCN-based node classification.

Experiments on four CTU-13 scenarios and the NCC-2 dataset demonstrate that RIGS maintains competitive botnet detection performance while reducing computational and memory costs. Although RIGS does not consistently achieve the highest classification score on every dataset or metric, the results indicate that its main advantage lies in providing a favorable balance among classification performance, computational efficiency, and structural preservation. In particular, the structural preservation analysis shows that RD favors highly connected nodes and global connectivity, whereas IFFST-PR better retains local clustering and community information. By combining these complementary properties, RIGS achieves low degree-distribution discrepancy while maintaining high connectivity, community consistency, and community coverage.

From the computational perspective, RIGS limits GNN computation to sampled subgraphs of bounded size and therefore reduces the computational scope of message passing on large graphs. Although the IFFST-PR component introduces a one-time PageRank preprocessing cost, the computed PageRank scores are reused throughout subsequent sampling iterations. The end-to-end evaluation shows that RIGS retains favorable computational efficiency after this preprocessing overhead is included, supporting its applicability to large-scale graph-based botnet detection.

Several limitations remain and provide directions for future research. First, the current study focuses on static communication graphs and therefore does not explicitly model the temporal evolution of botnet behavior. Future work will investigate dynamic graph modeling to capture time-varying communication patterns. Second, severe class imbalance and the limited number of botnet nodes in several scenarios may increase the variability in minority-class performance metrics; therefore, more robust learning and evaluation strategies for extremely imbalanced graph data should be explored. Third, the hybrid coefficient α is fixed after sensitivity analysis, and an adaptive strategy may better accommodate graphs with different structural characteristics.

In addition, practical botnet detection systems may operate in adversarial, encrypted, privacy-sensitive, and heterogeneous network environments. Adversaries may deliberately perturb traffic features or communication structures, potentially reducing the reliability of graph-based detection. Similar concerns regarding adversarial manipulation and security robustness have also been investigated in other data-security domains. For example, Lin et al. [34] analyzed attacks against a video cryptosystem and proposed corresponding security improvements. Although multimedia cryptography and graph-based botnet detection address different security layers, this study further highlights the importance of maintaining reliable security functionality under potentially adversarial conditions. Future work will therefore investigate robust graph learning, privacy-preserving feature extraction, and the applicability of RIGS to encrypted and heterogeneous communication environments.

## Figures and Tables

**Figure 1:**
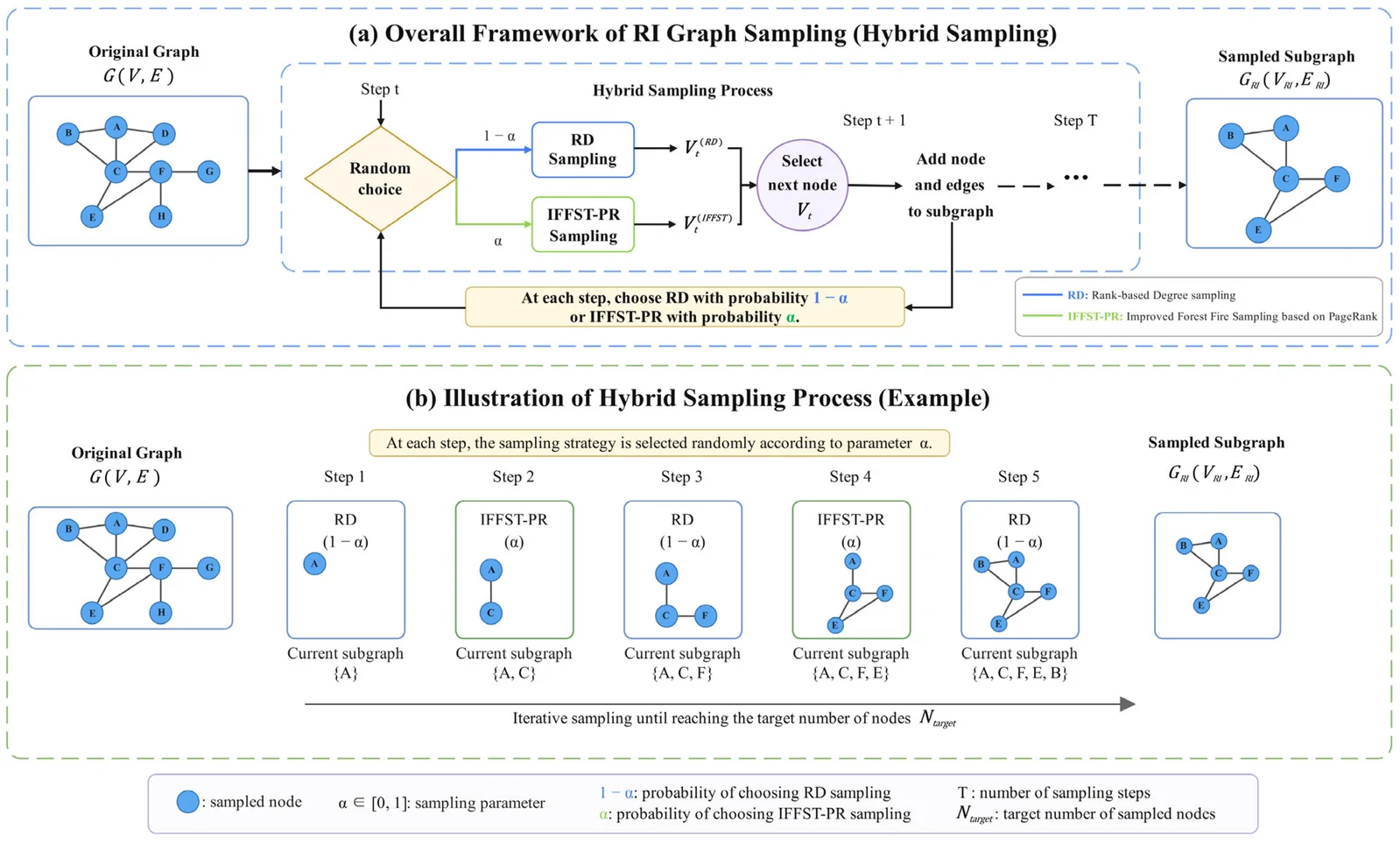
Overall framework of RI Graph Sampling. (**a**) Hybrid sampling framework integrating RD and IFFST-PR. At each sampling step, RD is selected with probability 1−α, whereas IFFST-PR is selected with probability α. (**b**) Illustration of the iterative hybrid sampling process, in which the sampled subgraph is progressively expanded until the predefined target number of sampled nodes is reached.

**Figure 2:**
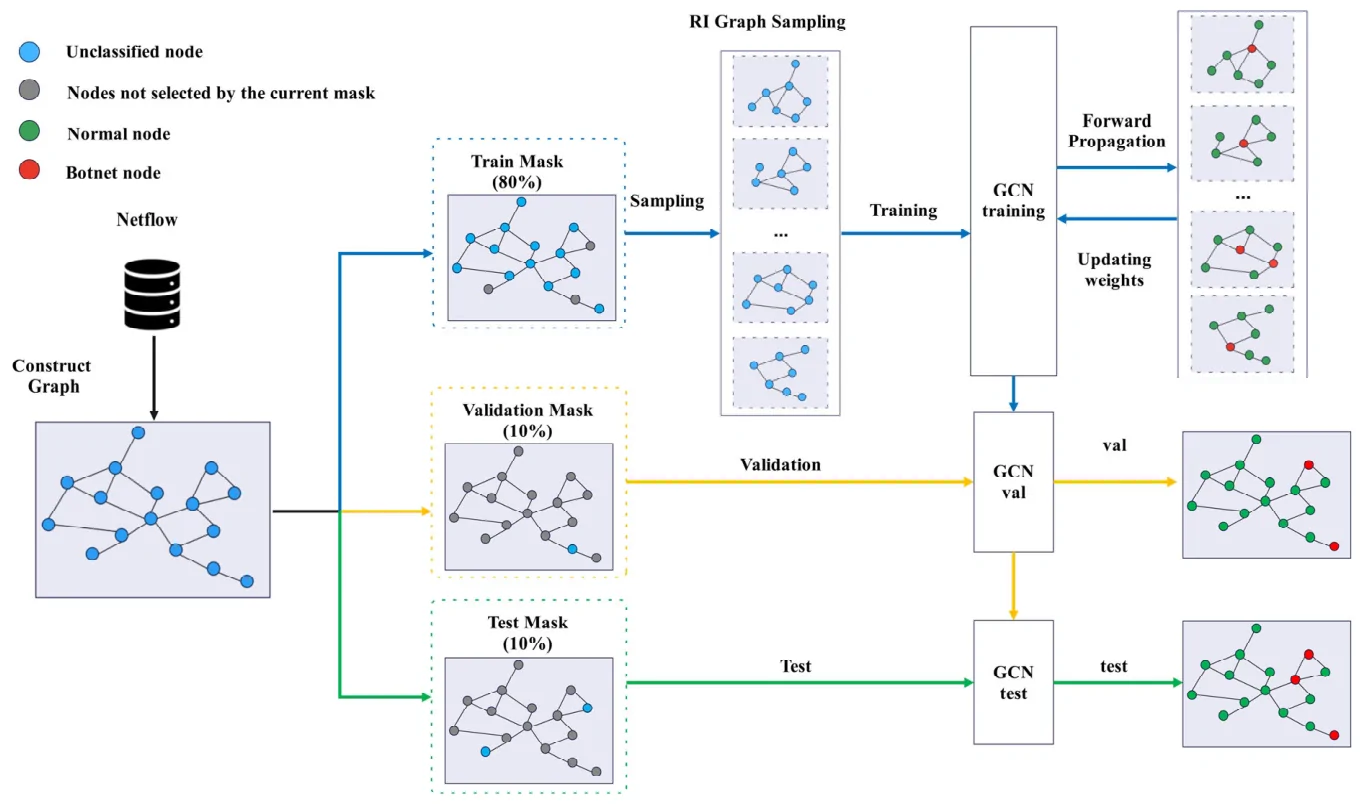
RI Graph Sampling-Based Botnet Detection Framework.

**Figure 3:**
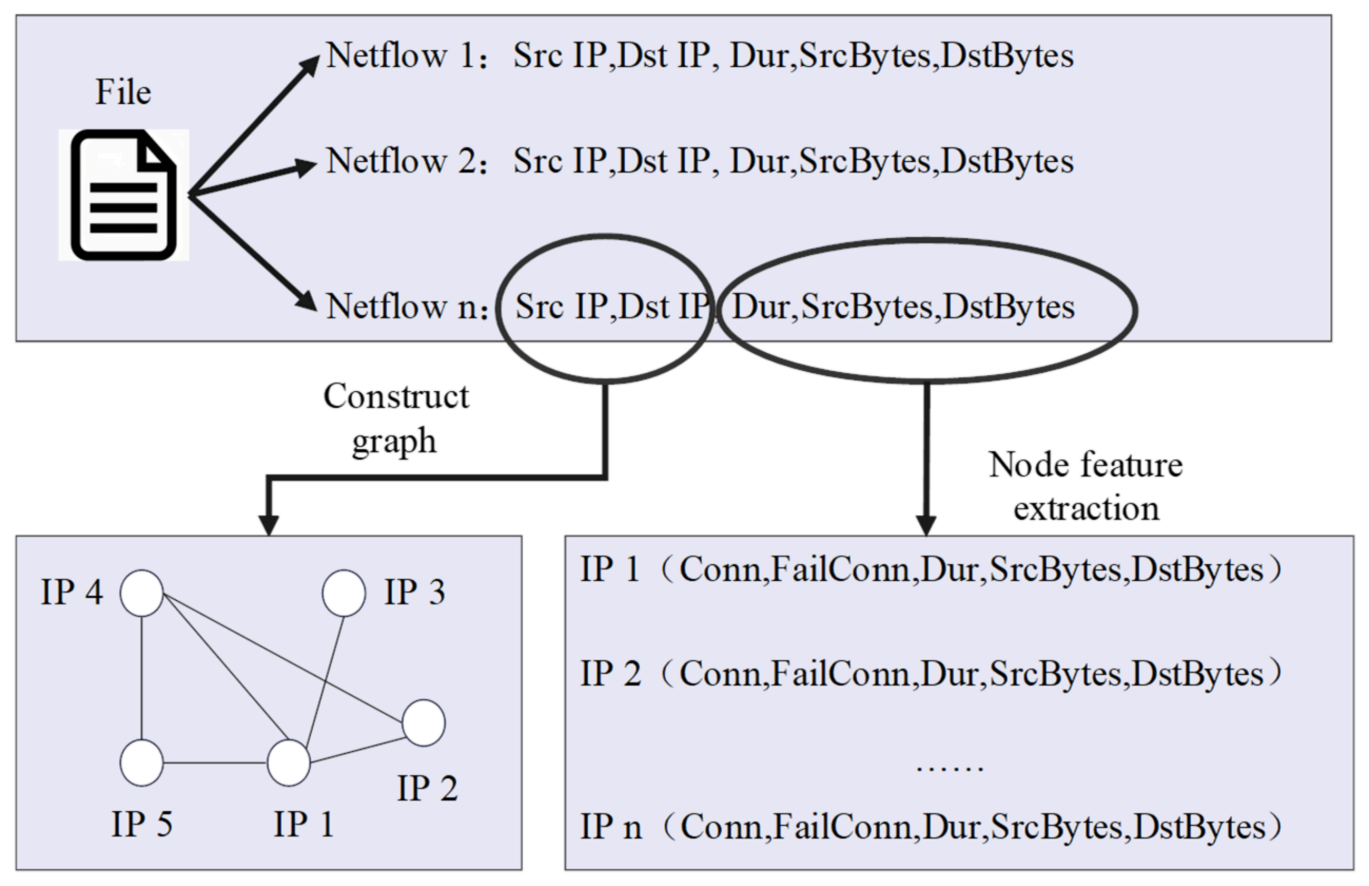
Graph Construction Flowchart.

**Figure 4:**
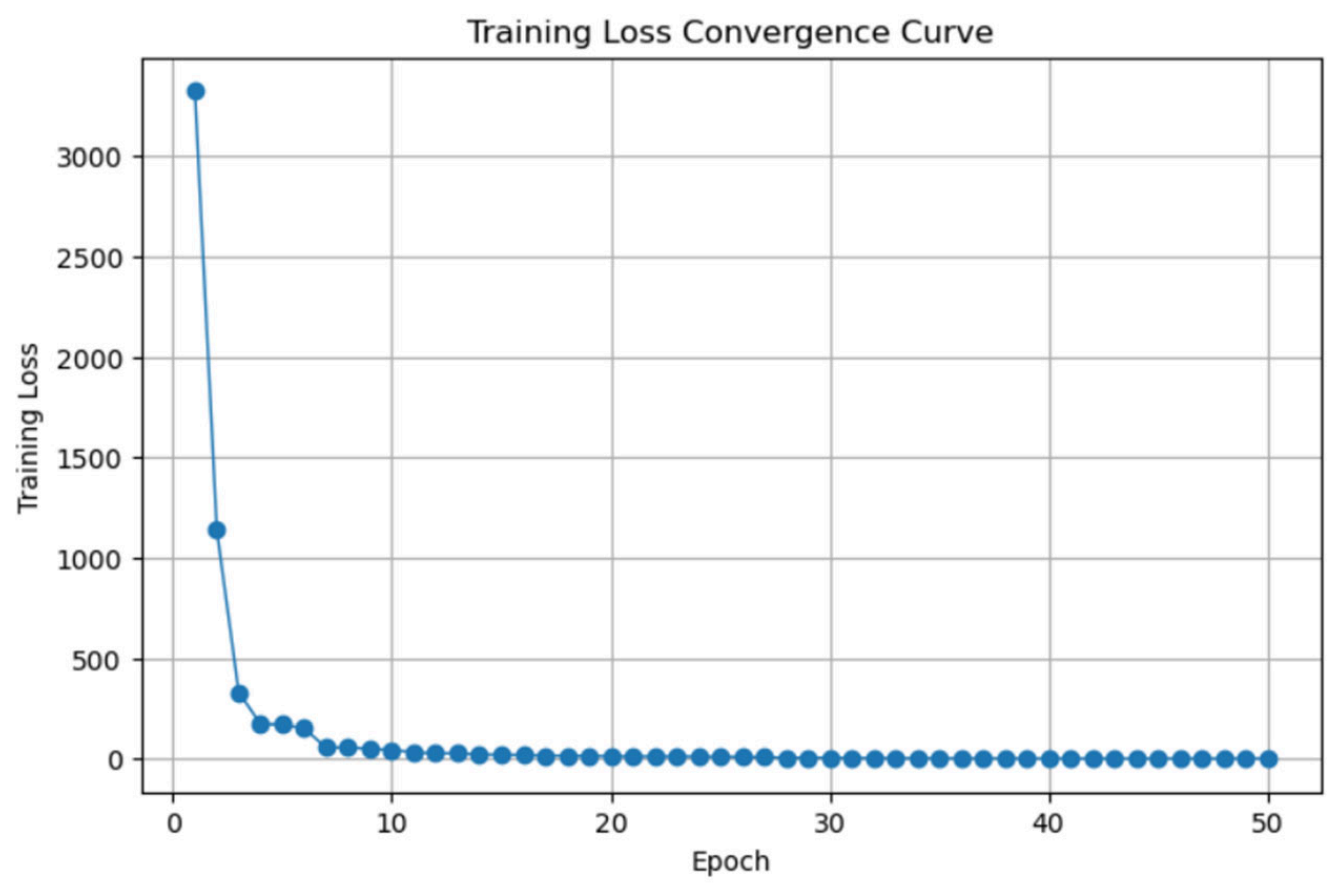
Training loss convergence curve of the RI Graph Sampling Model.

**Figure 5:**
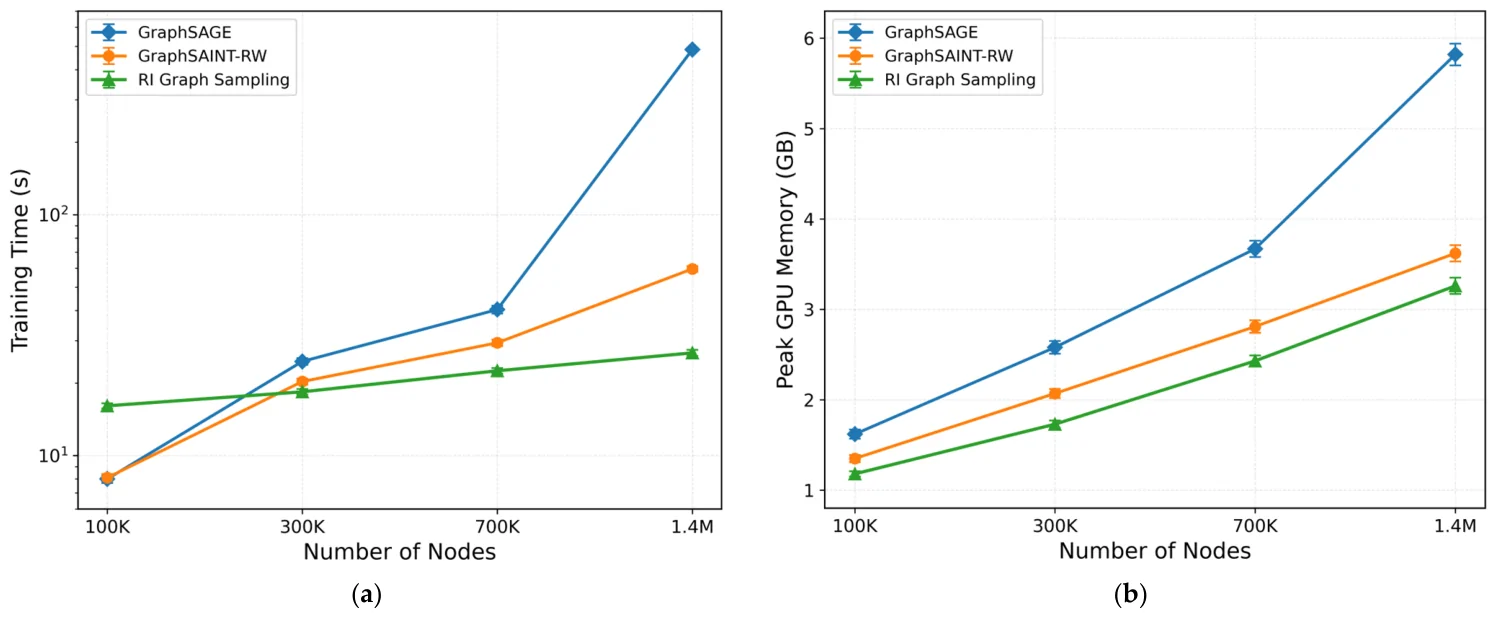
Computational scalability comparison under different NCC-2 graph sizes: (**a**) training time; (**b**) peak GPU memory consumption.

**Figure 6:**
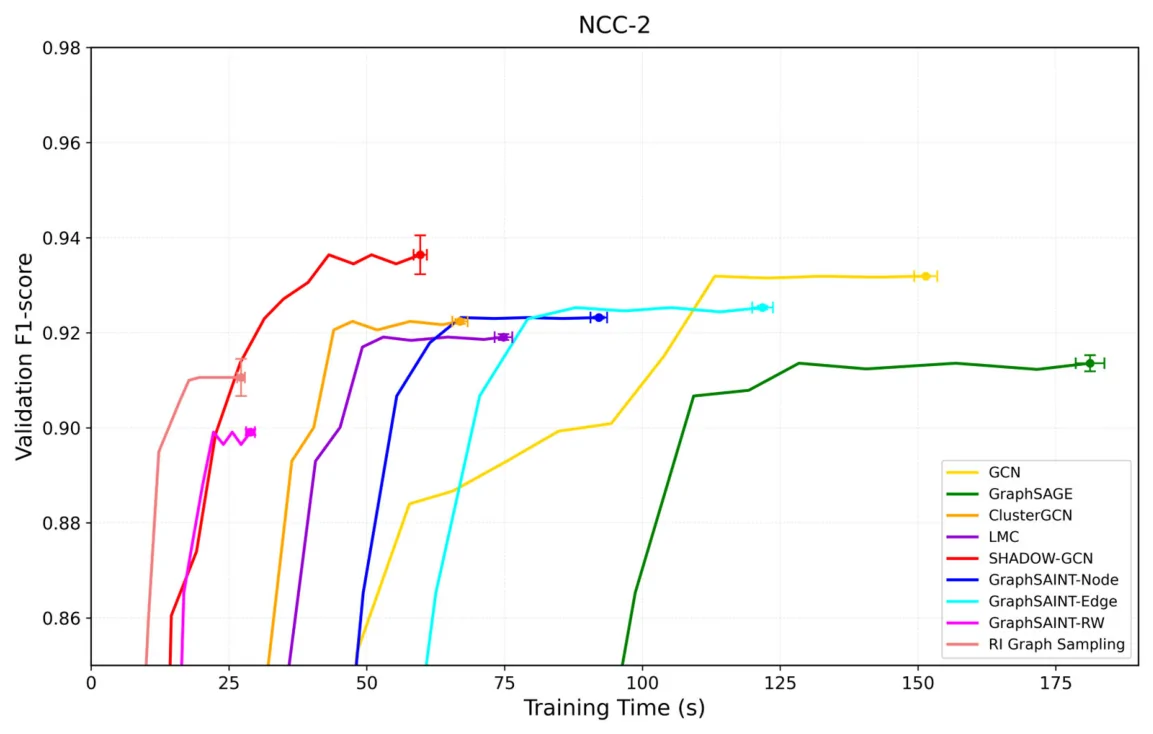
F1-Score convergence of RI Graph Sampling with other advanced methods on the NCC-2 dataset.

**Figure 7:**
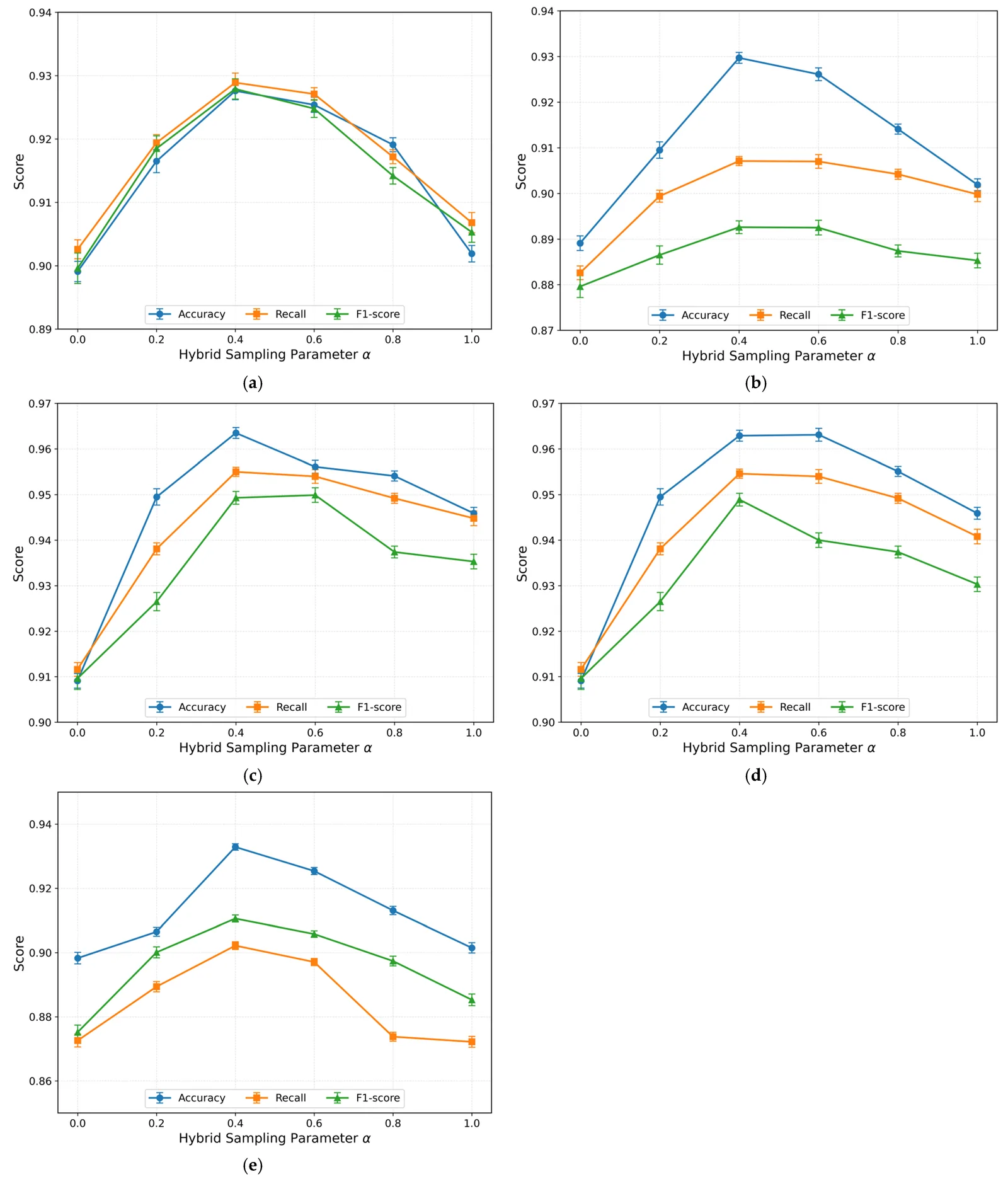
Sensitivity analysis of the hybrid sampling parameter α for RI Graph Sampling on five datasets: (**a**) Neris; (**b**) Rbot_1; (**c**) Rbot_2; (**d**) NSIS.ay; (**e**) NCC-2.

**Figure 8:**
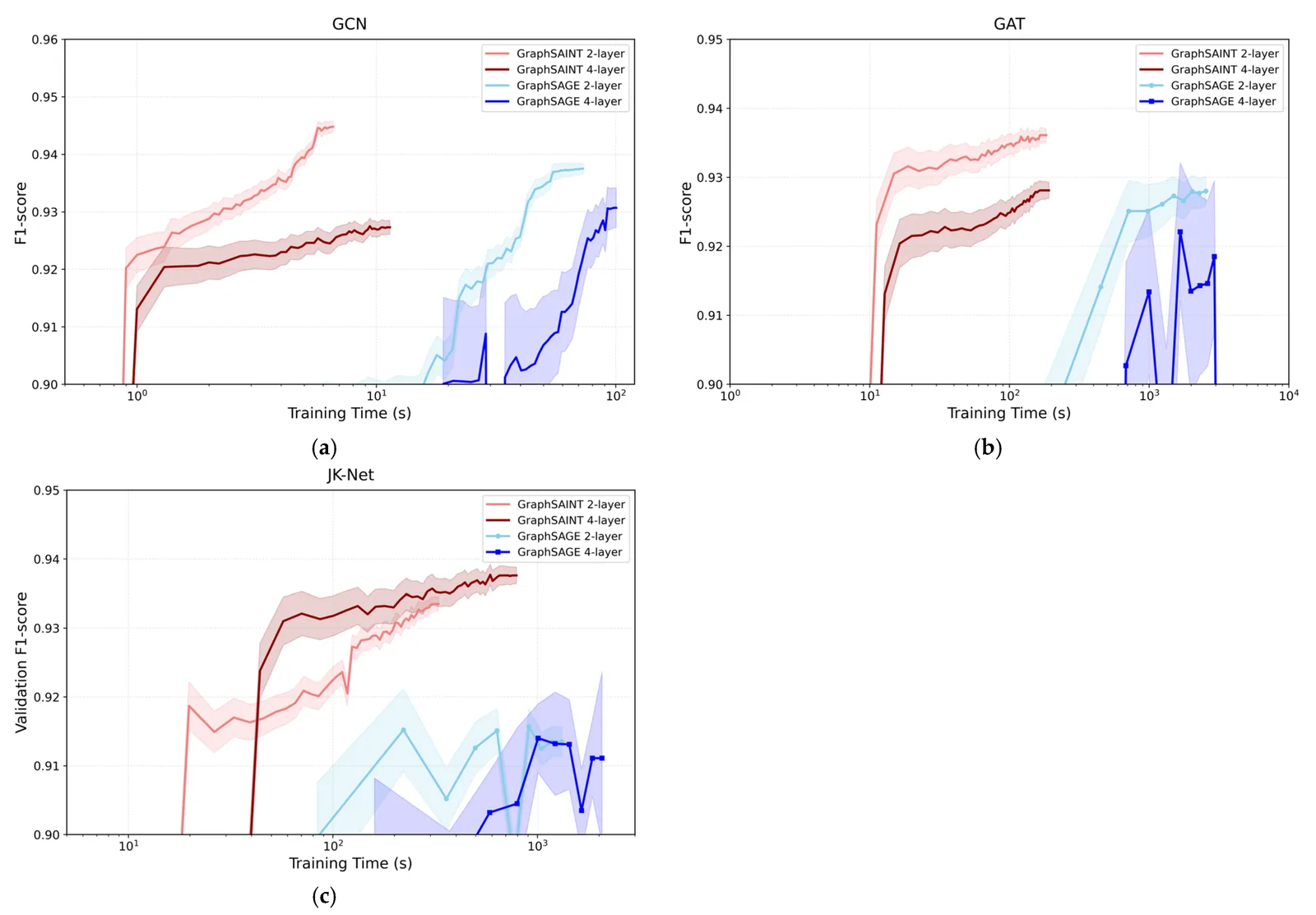
Performance comparison of GraphSAGE and GraphSAINT framework. (**a**) Comparison of the two sampling frameworks in GCN model; (**b**) Comparison of the two sampling frameworks in GAT model; (**c**) Comparison of the two sampling frameworks in JK-net model.

**Figure 9:**
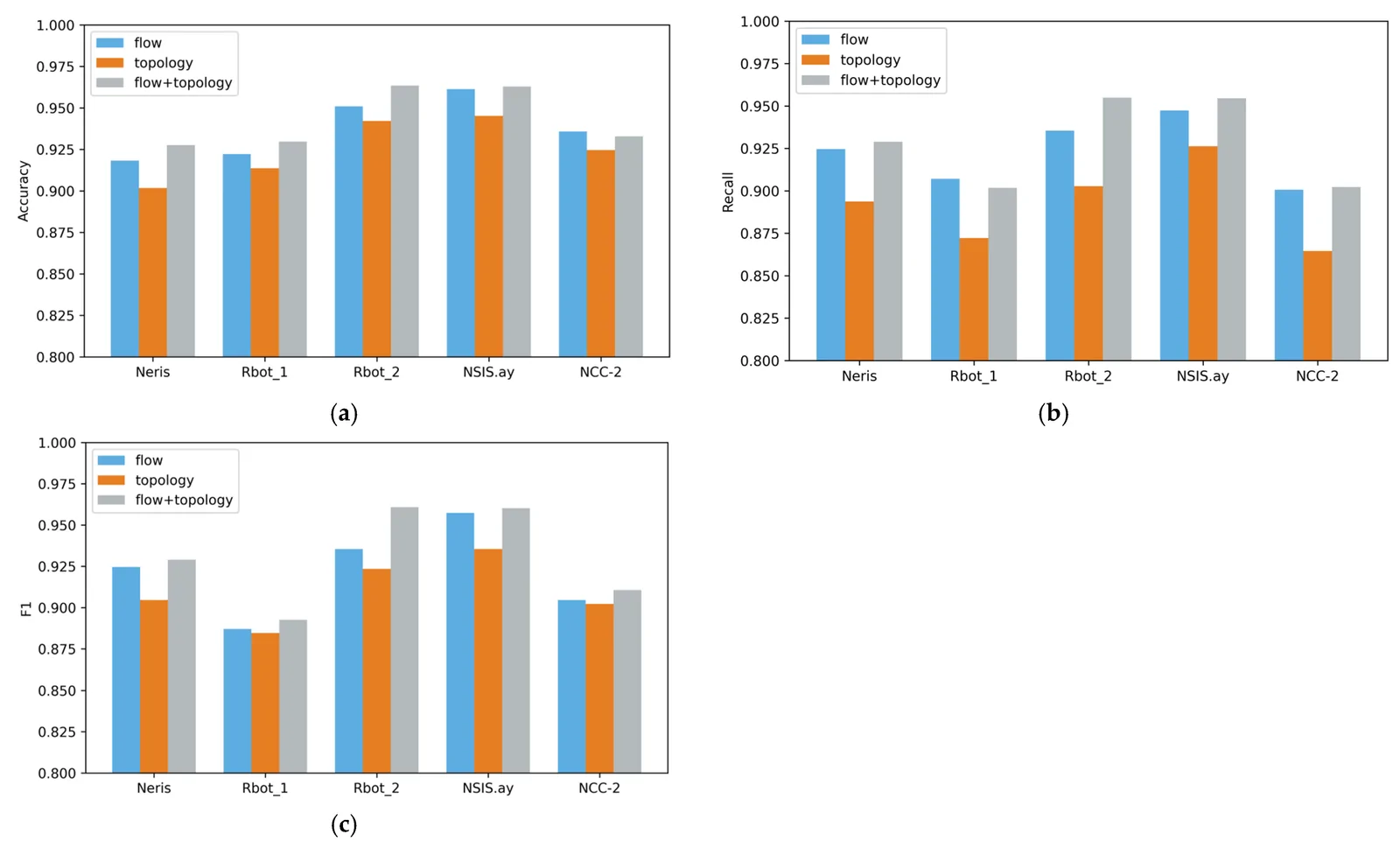
Ablation experiment of RI Graph Sampling flow feature and topological feature. (**a**) Comparison of ACC in flow feature and topology feature ablation experiments; (**b**) Comparison of Recall in flow feature and topology feature ablation experiments; (**c**) Comparison of F1-score in flow feature and topology feature ablation experiments.

**Table 1:** Experimental environment configuration.

Experimental Platform	Configuration
Operating System	Windows 11 24H2
CPU	Intel i7-10750H
GPU	NVIDIA GeForce RTX 2080 super
Memory	32.0 GB
IDE Tool	PyCharm Community Edition 2024.2 (64-bit)
Programming Language	Python 3.11
Deep Learning Framework	PyTorch 2.3.0 + cu121, torchvision 0.18.0 + cu121

**Table 2:** Data information of each scenario in the CTU-13 dataset.

Scenario	Duration/h	Packets	Netflows	Size/GB	Bot
1	6.15	71,971,482	2,824,637	52.0	Neris
2	4.21	71,851,300	1,808,123	60.0	Neris
3	66.85	167,730,395	4,710,639	121.0	Rbot
4	4.21	62,089,135	1,121,077	53.0	Rbot
5	11.63	4,481,167	129,833	37.6	Virut
6	2.18	38,764,357	558,920	30.0	Menti
7	0.38	7,467,139	114,078	5.8	Sogou
8	19.5	155,207,799	2,954,231	123.0	Murlo
9	5.18	115,415,321	2,753,885	94.0	Neris
10	4.75	90,389,782	1,309,792	73.0	Rbot
11	0.26	6,337,202	107,252	5.2	Rbot
12	1.21	13,212,268	325,472	8.3	NSIS.ay
13	16.36	50,888,256	1,925,150	34.0	Virut

**Table 3:** Data statistics of each scenario in the NCC-2 dataset.

Scenario	Duration/h	Netflows	Size/GB
1	8	4,895,158	0.62
2	8	5,998,133	0.74
3	8	3,885,792	0.52
all	8	14,779,083	1.95

**Table 4:** CTU-13 dataset after graph conversion.

Id	Bot	Nodes	Edges	Botnet Nodes
1	Neris	499,301	1,023,630	1
2	Neris	359,837	710,515	1
3	Rbot	354,887	773,662	1
4	Rbot	150,799	321,227	1
5	Virut	30,641	63,364	1
6	Menti	84,252	179,718	1
7	Sogou	28,141	58,254	1
8	Murlo	320,850	687,012	1
9	Neris	300,301	682,803	10
10	Rbot	161,397	358,122	10
11	Rbot	30,802	63,378	3
12	NSIS.ay	71,307	144,910	3
13	Virut	261,977	554,617	1

**Table 5:** NCC-2 dataset after graph conversion.

Id	Nodes	Edges	Botnet Nodes
1	690,209	1,458,672	17
2	831,477	1,735,051	10
3	442,857	1,007,426	17
all	1,398,418	3,032,846	17

**Table 6:** Datasets Selected for Experiments.

Id	Bot	Nodes	Edges	Botnet Nodes
1	Neris	300,301	682,803	10
2	Rbot_1	161,397	358,122	10
3	Rbot_2	30,802	63,378	3
4	NSIS.ay	71,307	144,910	3
5	NCC-2	1,398,418	3,032,846	17

**Table 7:** Node Distribution in the Training, Validation, and Test Splits.

Id	Bot	Split	Nodes	Botnet Nodes
1	Neris	Train	240,241	8
		Validation	30,030	1
		Test	30,030	1
2	Rbot_1	Train	129,117	8
		Validation	16,140	1
		Test	16,140	1
3	Rbot_2	Train	24,642	1
		Validation	3080	1
		Test	3080	1
4	NSIS.ay	Train	57,045	1
		Validation	7131	1
		Test	7131	1
5	NCC-2	Train	1,118,734	13
		Validation	139,842	2
		Test	139,842	2

**Table 8:** Definitions and Aggregation Rules of Node Features.

Feature	Grouped by	Definition
Conn	Source IP	Number of valid flows initiated by the source IP
FailConn	Source IP	Number of flows with zero duration
Dur	Source IP	Mean duration of flows with duration >0
SrcBytes	Source IP	Mean number of transmitted bytes
DstBytes	Destination IP	Mean number of received bytes

**Table 9:** Experimental configuration and hyperparameter settings.

Category	Parameter	Setting
RIGS	Target subgraph size $N_{target}$	6000
RIGS	Hybrid sampling coefficient α	0.4
RD	Initial node set r	10
RD	Number of top-ranked neighbors k	5
IFFST-PR	Forest fire propagation probability p	0.4
PageRank	Damping coefficient s	0.85
PageRank	Maximum number of iterations	100
GCN	Number of layers	2
GCN	Hidden layer dimension	256
Training	Loss function	Focal Loss
Training	Focusing parameter γ	2
Training	Balancing factor $\alpha_{t}$	0.75
Training	Optimizer	Adam
Training	Learning rate	0.001
Training	Dropout rate	0.2
Training	Number of training epochs	50
Training	Number of sampled subgraphs per epoch	50
Reproducibility	Random seeds	42–46 (5 seeds)

**Table 10:** Class Distribution of NCC-2 at Different Graph Sizes.

Graph Size	Botnet Nodes	Normal Nodes	Botnet Ratio
100 K	17	99,983	0.0170%
300 K	17	299,983	0.0057%
700 K	17	699,983	0.0024%
1.4 M	17	1,398,401	0.0012%

**Table 11:** Hyperparameter Settings of the Baseline Methods.

Method	Layers	Hyperparameters
GCN	GCN/2	Full-graph training; hidden dimension = 256
GraphSAGE	GraphSAGE/2	Aggregator = Mean; fan-out = [25, 10]; batch size = 512
ClusterGCN	GCN/2	Number of partitions = 1500; clusters per batch = 5
LMC	GCN/2	Number of partitions = 1500; clusters per mini-batch = 5; 1-hop forward and backward message compensation
SHADOW-GCN	GCN/2	Extraction method = PPR; subgraph node budget = 200
GraphSAINT-Node	GCN/2	Node budget = 6000; sampler batch size = 32
GraphSAINT-Edge	GCN/2	Edge budget = 3500; sampler batch size = 32
GraphSAINT-RW	GCN/2	Number of roots = 4000; walk length = 7; sampler batch size = 32

**Table 12:** ACC Comparison of RI Graph Sampling and Other Advanced Methods.

Model	Neris	Rbot_1	Rbot_2	NSIS.ay	NCC-2
GCN	0.9150 ± 0.0003	0.9034 ± 0.0001	0.9430 ± 0.0006	0.9421 ± 0.0004	0.9327 ± 0.0003
GraphSAGE	0.9330 ± 0.0011	0.9380 ± 0.0004	0.9574 ± 0.0009	0.9566 ± 0.0006	0.9143 ± 0.0011
ClusterGCN	0.9035 ± 0.0005	0.9002 ± 0.0004	0.9184 ± 0.0007	0.9180 ± 0.0007	0.9051 ± 0.0006
LMC	0.9160 ± 0.0006	0.9166 ± 0.0005	0.9411 ± 0.0008	0.9407 ± 0.0007	0.9119 ± 0.0005
SHADOW-GCN	**0.9357 ± 0.0038**	**0.9389 ± 0.0009**	0.9616 ± 0.0052	0.9608 ± 0.0061	**0.9370 ± 0.0019**
GraphSAINT-Node	0.9272 ± 0.0003	0.9338 ± 0.0002	0.9658 ± 0.0006	0.9652 ± 0.0008	0.9235 ± 0.0003
GraphSAINT-Edge	0.9318 ± 0.0005	0.9359 ± 0.0003	0.9565 ± 0.0005	0.9561 ± 0.0005	0.9312 ± 0.0002
GraphSAINT-RW	0.9264 ± 0.0002	0.9295 ± 0.0004	**0.9692 ± 0.0008**	**0.9686 ± 0.0006**	0.9014 ± 0.0003
Ours	0.9276 ± 0.0042	0.9297 ± 0.0068	0.9635 ± 0.0077	0.9629 ± 0.0075	0.9329 ± 0.0040

**Table 13:** Precision Comparison of RI Graph Sampling and Other Advanced Methods.

Model	Neris	Rbot_1	Rbot_2	NSIS.ay	NCC-2
GCN	0.9021 ± 0.0013	0.9034 ± 0.0009	0.9087 ± 0.0015	0.9082 ± 0.0017	0.9161 ± 0.0009
GraphSAGE	0.9108 ± 0.0015	0.9187 ± 0.0016	0.9075 ± 0.0026	0.9061 ± 0.0024	0.9109 ± 0.0039
ClusterGCN	0.9096 ± 0.0010	0.9051 ± 0.0019	0.9338 ± 0.0018	0.9327 ± 0.0016	0.9398 ± 0.0017
LMC	**0.9369 ± 0.0013**	**0.9372 ± 0.0015**	0.9452 ± 0.0019	0.9448 ± 0.0018	0.9227 ± 0.0015
SHADOW-GCN	0.9308 ± 0.0041	0.9349 ± 0.0064	**0.9527 ± 0.0014**	**0.9521 ± 0.0062**	0.9345 ± 0.0011
GraphSAINT-Node	0.9247 ± 0.0015	0.9318 ± 0.0011	0.9464 ± 0.0015	0.9459 ± 0.0021	0.9283 ± 0.0008
GraphSAINT-Edge	0.9219 ± 0.0013	0.9020 ± 0.0015	0.9431 ± 0.0014	0.9426 ± 0.0012	0.9328 ± 0.0011
GraphSAINT-RW	0.9258 ± 0.0007	0.9271 ± 0.0010	0.9398 ± 0.0014	0.9406 ± 0.0020	**0.9352 ± 0.0018**
Ours	0.9280 ± 0.0024	0.9212 ± 0.0013	0.9443 ± 0.0015	0.9438 ± 0.0016	0.9278 ± 0.0012

**Table 14:** Recall Comparison of RI Graph Sampling and Other Advanced Methods.

Model	Neris	Rbot_1	Rbot_2	NSIS.ay	NCC-2
GCN	0.9187 ± 0.0006	0.9153 ± 0.0002	0.9511 ± 0.0004	0.9504 ± 0.0007	0.9169 ± 0.0005
GraphSAGE	0.9326 ± 0.0009	0.9373 ± 0.0012	0.9446 ± 0.0005	0.9441 ± 0.0016	0.9145 ± 0.0013
ClusterGCN	0.8809 ± 0.0006	0.8819 ± 0.0005	0.8946 ± 0.0006	0.8940 ± 0.0005	0.9043 ± 0.0007
LMC	0.9061 ± 0.0007	0.9099 ± 0.0006	0.9275 ± 0.0008	0.9269 ± 0.0009	0.9148 ± 0.0007
SHADOW-GCN	0.9293 ± 0.0035	0.9400 ± 0.0007	0.9538 ± 0.0034	0.9531 ± 0.0059	0.9268 ± 0.0043
GraphSAINT-Node	0.9202 ± 0.0003	**0.9439 ± 0.0005**	0.9587 ± 0.0008	0.9582 ± 0.0004	**0.9371 ± 0.0004**
GraphSAINT-Edge	**0.9376 ± 0.0007**	0.9374 ± 0.0002	0.9539 ± 0.0004	0.9535 ± 0.0003	0.9368 ± 0.0002
GraphSAINT-RW	0.9362 ± 0.0003	0.9324 ± 0.0004	**0.9685 ± 0.0007**	**0.9679 ± 0.0006**	0.9018 ± 0.0007
Ours	0.9289 ± 0.0060	0.9071 ± 0.0045	0.9550 ± 0.0042	0.9546 ± 0.0041	0.9022 ± 0.0052

**Table 15:** F1-Score Comparison of RI Graph Sampling and Other Advanced Methods.

Model	Neris	Rbot_1	Rbot_2	NSIS.ay	NCC-2
GCN	0.9099 ± 0.0004	0.9035 ± 0.0003	0.9291 ± 0.0005	0.9285 ± 0.0006	0.9162 ± 0.0001
GraphSAGE	0.9213 ± 0.0003	0.9276 ± 0.0001	0.9029 ± 0.0011	0.9024 ± 0.0008	0.9124 ± 0.0017
ClusterGCN	0.8947 ± 0.0001	0.8930 ± 0.0008	0.9135 ± 0.0006	0.9126 ± 0.0005	0.9214 ± 0.0005
LMC	0.9120 ± 0.0005	0.9116 ± 0.0007	0.9360 ± 0.0008	0.9355 ± 0.0007	0.9184 ± 0.0006
SHADOW-GCN	0.9297 ± 0.0006	0.9371 ± 0.0030	**0.9529 ± 0.0046**	0.9523 ± 0.0069	**0.9303 ± 0.0041**
GraphSAINT-Node	0.9221 ± 0.0006	**0.9375 ± 0.0003**	0.9522 ± 0.0004	0.9517 ± 0.0009	0.9232 ± 0.0002
GraphSAINT-Edge	0.9294 ± 0.0004	0.9184 ± 0.0006	0.9482 ± 0.0005	0.9477 ± 0.0004	0.9253 ± 0.0004
GraphSAINT-RW	**0.9307 ± 0.0001**	0.9294 ± 0.0003	0.9536 ± 0.0004	**0.9538 ± 0.0008**	0.8991 ± 0.0006
Ours	0.9279 ± 0.0058	0.8926 ± 0.0040	0.9493 ± 0.0054	0.9489 ± 0.0072	0.9106 ± 0.0039

**Table 16:** MCC Comparison of RI Graph Sampling and Other Advanced Methods.

Model	Neris	Rbot_1	Rbot_2	NSIS.ay	NCC-2
GCN	0.8206 ± 0.0006	0.8186 ± 0.0003	0.8588 ± 0.0010	0.8576 ± 0.0008	0.8330 ± 0.0006
GraphSAGE	0.8431 ± 0.0020	0.8558 ± 0.0009	0.8513 ± 0.0015	0.8494 ± 0.0013	0.8254 ± 0.0021
ClusterGCN	0.7900 ± 0.0009	0.7867 ± 0.0008	0.8275 ± 0.0012	0.8258 ± 0.0012	0.8434 ± 0.0015
LMC	0.8424 ± 0.0011	0.8467 ± 0.0010	0.8725 ± 0.0014	0.8715 ± 0.0013	0.8375 ± 0.0012
SHADOW-GCN	0.8601 ± 0.0061	0.8749 ± 0.0020	0.9065 ± 0.0095	0.9052 ± 0.0108	0.8613 ± 0.0041
GraphSAINT-Node	0.8449 ± 0.0007	**0.8756 ± 0.0006**	0.9050 ± 0.0012	0.9040 ± 0.0014	0.8654 ± 0.0007
GraphSAINT-Edge	0.8594 ± 0.0010	0.8387 ± 0.0006	0.8969 ± 0.0011	0.8960 ± 0.0010	**0.8696 ± 0.0005**
GraphSAINT-RW	**0.8619 ± 0.0006**	0.8595 ± 0.0008	**0.9078 ± 0.0013**	**0.9081 ± 0.0012**	0.8363 ± 0.0007
Ours	0.8565 ± 0.0015	0.8282 ± 0.0017	0.8992 ± 0.0018	0.8983 ± 0.0015	0.8296 ± 0.0022

**Table 17:** End-to-End Computational Cost on the NCC-2 dataset.

Model	Preprocessing Time (s)	Sampling Time (s)	Training Time (s)	Total Time (s)	Peak GPU Memory (GB)
GCN	0	0	151.4 ± 2.1	151.4 ± 2.1	6.31 ± 0.10
GraphSAGE	0	0	181.2 ± 2.6	181.2 ± 2.6	4.82 ± 0.12
ClusterGCN	8.6 ± 0.4	5.7 ± 0.3	66.9 ± 1.4	81.2 ± 1.5	4.36 ± 0.10
LMC	5.2 ± 0.3	9.4 ± 0.4	74.8 ± 1.6	89.4 ± 1.7	3.91 ± 0.11
SHADOW-GCN	3.8 ± 0.2	6.3 ± 0.3	59.7 ± 1.2	69.8 ± 1.3	3.53 ± 0.10
GraphSAINT-Node	4.6 ± 0.2	9.7 ± 0.4	92.1 ± 1.5	106.4 ± 1.6	3.79 ± 0.09
GraphSAINT-Edge	5.1 ± 0.2	10.2 ± 0.5	121.8 ± 1.9	137.1 ± 2.0	3.95 ± 0.09
GraphSAINT-RW	4.3 ± 0.2	9.1 ± 0.3	28.9 ± 0.8	42.3 ± 0.9	3.62 ± 0.09
Ours	7.8 ± 0.3	5.9 ± 0.3	**27.2 ± 0.7**	**40.9 ± 0.8**	**3.26 ± 0.0** **9**

**Table 18:** Comparison of ACC in RI Graph Sampling Ablation Experiments.

Model	Neris	Rbot_1	Rbot_2	NSIS.ay	NCC-2
RD	0.8962 ± 0.0051	0.9155 ± 0.0047	0.9528 ± 0.0059	0.9590 ± 0.0043	**0.9338 ± 0.0056**
IFFST-PR	0.9046 ± 0.0048	0.9189 ± 0.0054	0.9363 ± 0.0062	0.9572 ± 0.0046	0.9320 ± 0.0050
Ours	**0.9276 ± 0.0042**	**0.9297 ± 0.0068**	**0.9635 ± 0.0077**	**0.9629 ± 0.0075**	0.9329 ± 0.0040

**Table 19:** Comparison of Recall in RI Graph Sampling Ablation Experiments.

Model	Neris	Rbot_1	Rbot_2	NSIS.ay	NCC-2
RD	0.9183 ± 0.0055	**0.9162 ± 0.0049**	0.9489 ± 0.0057	**0.9601 ± 0.0044**	0.9006 ± 0.0058
IFFST-PR	0.9091 ± 0.0047	0.9019 ± 0.0053	0.9386 ± 0.0051	0.9534 ± 0.0048	**0.9309 ± 0.0056**
Ours	**0.9289 ± 0.0060**	0.9071 ± 0.0045	**0.9550 ± 0.0042**	0.9546 ± 0.0041	0.9022 ± 0.0052

**Table 20:** Comparison of F1-Score in RI Graph Sampling Ablation Experiments.

Model	Neris	Rbot_1	Rbot_2	NSIS.ay	NCC-2
RD	0.9071 ± 0.0050	**0.9123 ± 0.0046**	0.9306 ± 0.0058	0.9396 ± 0.0045	0.9175 ± 0.0053
IFFST-PR	0.9047 ± 0.0046	0.9103 ± 0.0051	0.9379 ± 0.0055	0.9323 ± 0.0047	**0.9** **3** **12 ± 0.0058**
Ours	**0.9279 ± 0.0058**	0.8926 ± 0.0040	**0.9493 ± 0.0054**	**0.9489 ± 0.0072**	0.9106 ± 0.0039

**Table 21:** Structural Preservation Comparison of Different Graph Sampling Methods.

Model	Avg Degree	KS	LCC Ratio	Avg CC	NMI	Coverage
Original Full Graph	4.3375	0	1	0.0482	1	1
GraphSAINT-Node	7.5152 ± 0.1843	0.1437 ± 0.0008	0.9916 ± 0.0001	0.0129 ± 0.0001	0.0146 ± 0.0002	0.6523 ± 0.0016
GraphSAINT-Edge	**5.1832 ± 0.1265**	0.1643 ± 0.0009	0.9959 ± 0.0001	0.0014 ± 0.0000	0.0150 ± 0.0002	0.6364 ± 0.0018
GraphSAINT-RW	5.8187 ± 0.1538	0.1721 ± 0.0011	**0.9996 ± 0.0000**	0.0037 ± 0.0001	0.0098 ± 0.0001	0.6872 ± 0.0014
RD Graph Sampling	16.3925 ± 0.6247	0.3357 ± 0.0026	0.9992 ± 0.0001	0.0046 ± 0.0001	0.6066 ± 0.0021	0.6303 ± 0.0019
IFFST-PR Graph Sampling	3.4106 ± 0.0986	0.1397 ± 0.0007	0.8602 ± 0.0018	**0.0428 ± 0.0002**	0.7215 ± 0.0017	0.6879 ± 0.0013
RI Graph Sampling	8.6911 ± 0.2154	**0.0672 ± 0.0004**	0.9990 ± 0.0001	0.0352 ± 0.0002	**0.8124 ± 0.0012**	**0.6909 ± 0.0011**

## Data Availability

CTU-13 dataset: https://www.stratosphereips.org/datasets-ctu13 (accessed on 9 August 2026). NCC-2 dataset: https://data.mendeley.com/datasets/8dpt85jrhp/2 (accessed on 9 August 2026).
